# Derivation of paediatric blood pressure percentiles from electronic health records

**DOI:** 10.1016/j.ebiom.2023.104885

**Published:** 2023-11-19

**Authors:** Mark M. Mitsnefes, Mitchell Maltenfort, Michelle R. Denburg, Joseph T. Flynn, Julia Schuchard, Bradley B. Dixon, Hiren P. Patel, Donna Claes, Kimberley Dickinson, Yong Chen, Caroline Gluck, Mary Leonard, Priya S. Verghese, Christopher B. Forrest

**Affiliations:** a Cincinnati Children's Hospital Medical Center, Department of Pediatrics, University of Cincinnati, OH, USA; b Children's Hospital of Philadelphia, Applied Clinical Research Center, Philadelphia, PA, USA; c Division of Nephrology, Children's Hospital of Philadelphia, Philadelphia, PA, USA; d Department of Pediatrics, Perelman School of Medicine at the University of Pennsylvania: Philadelphia, PA, USA; e Department of Biostatistics, Epidemiology and Informatics, Perelman School of Medicine at the University of Pennsylvania, Philadelphia, PA, USA; f Division of Nephrology, Department of Pediatrics, University of Washington School of Medicine, Seattle Children's Hospital, Seattle, WA, USA; g Department of Pediatrics, Children's Hospital Colorado, University of Colorado School of Medicine, Denver, CO, USA; h Nationwide Children's Hospital, Columbus, OH, USA; i Nemours/Alfred I. DuPont Hospital for Children, Wilmington, DE, USA; j Stanford University, Stanford, CA, USA; k Ann & Robert H Lurie Children's Hospital, Northwestern Feinberg School of Medicine, Chicago, IL, USA

**Keywords:** Blood pressure references, Hypertension, Children, Paediatric, Electronic health record (EHR)

## Abstract

**Background:**

Identification of abnormal blood pressure (BP) in children requires normative data. We sought to examine the feasibility of using “real-world” office BP data obtained from electronic health records (EHR) to generate age-, sex- and height-specific BP percentiles for children.

**Methods:**

Using data collected 01/01/2009–8/31/2021 from eight large children's healthcare organisations in PEDSnet, we applied a mixed-effects polynomial regression model with random slopes to generate Z-scores and BP percentiles and compared them with currently used normative BP distributions published in the 2017 American Academy of Paediatrics (AAP) Clinical Practise Guidelines (CPG).

**Findings:**

We identified a study sample of 292,412 children (1,085,083 BP measurements), ages 3–17 years (53% female), with no chronic medical conditions, who were not overweight/obese and who were primarily seen for general paediatric care in outpatient settings. Approximately 45,000–75,000 children contributed data to each age category. The PEDSnet systolic BP percentile values were 1–4 mmHg higher than AAP CPG BP values across age-sex-height groups, with larger differences observed in younger children. Diastolic BP values were also higher in younger children; starting with age 7 years, diastolic BP percentile values were 1–3 mmHg lower than AAP CPG values. Cohen's Kappa was 0.90 for systolic BP, 0.66 for diastolic BP, and 0.80 overall indicating excellent agreement between PEDSnet and 2017 AAP CPG data for systolic BP and substantial agreement for diastolic BP.

**Interpretation:**

Our analysis indicates that real-word EHR data can be used to generate BP percentiles consistent with current clinical practise on BP management in children.

**Funding:**

Funding for this work was provided by the Preserving Kidney Function in Children with Chronic Kidney Disease (PRESERVE) study; Patient-Centred Outcomes Research Institute (PCORI) RD-2020C2020338 (Principal Investigator: Dr. Forrest; Co-Principal Investigator: Dr. Denburg).

**Research in context**

**Evidence before this study**

Numerous studies have shown that high blood pressure (BP) in childhood increases the risk for adult hypertension. Therefore, it is important to identify and treat children with elevated BP. Identification of abnormal BP in paediatric patients requires normative data. Data for current BP references were collected 30–50 years ago by auscultation and through BP research studies. We searched MEDLINE with the terms “normative BP references”, “paediatric”, “children”, “blood pressure percentiles”, “BP guidelines” published in English up to July 15, 2023.

**Added value of this study**

We generated age-, sex- and height-specific BP percentiles using BP measures from PEDSnet (pedsnet.org), a multi-institutional paediatric health system network that currently aggregates electronic health record (EHR) data from more than 8 million children. We identified a study sample study sample of 292,412 children (1,085,083 BP measurements) without evidence of overweight/obesity, chronic diseases, or BP-altering medication, estimated BP distributions for this study sample, and compared these distributions with historically generated BP percentiles values (2017 American Academy of Paediatrics Clinical Practise Guidelines, AAP CPG). Our statistical model incorporated random effect slopes for age and height Z-score to account for change in variability with age and height. The analysis demonstrated excellent agreement between PEDSnet and AAP CPG data for systolic BP and substantial agreement for diastolic BP and indicated that real-word EHR data can be used to generate BP percentiles consistent with current clinical practise on BP management in children.

**Implications of all the available evidence**

Our results indicate that generating empirical distributions of BP values using large samples from the EHR is feasible and cost-effective. The data could be efficiently updated on a periodic basis (e.g., once a decade) to reflect changes in US demographics or in clinical practise of BP management. If these EHR-derived distributions are confirmed, some young children who were previously diagnosed with high BP would be reclassified as having normal BP. Adopting EHR-derived percentiles may lead to changes in follow up, evaluation, referral to subspecialist and treatment.

## Introduction

Identification of abnormal blood pressure (BP) in paediatric patients requires normative data. Current normative BP values in children are based on empirical distributions of BP adjusted for age, sex, and height. The initial set of normative BP percentile estimates was published in 1977 as part of the first comprehensive report on evaluation, diagnosis and treatment of elevated BP in children.^1^ These estimates were replaced with newly derived data published in 1987 in the report of the second Task Force on BP control in children and further updated by the National High Blood Pressure Education Programme Working Group on high BP in 1996 and 2004 using additional BP measurements obtained from the National Health and Nutrition Examination Surveys (NHANES).^2^, ^3^, ^4^ These estimates were based upon BP readings obtained in 63,237 children from 83,091 visits across 11 large paediatric research studies. Because of rising rates of obesity in childhood and its impact on BP, the tables were further modified to exclude overweight children, resulting in a final dataset of 49,967 children.^5^ These tables are currently included in the most recent 2017 American Academy of Paediatrics (AAP) Clinical Practise Guidelines (CPG) on BP in children.^6^ Most of the data used in the latest analysis were collected from the 1970s–1990s, with the latest addition of approximately 1300 children from NHANES in 1999–2000.

Potential limitations of existing BP references include collection of the data through research studies with most of the data obtained 25–50 years ago; modest sample size of the dataset, particularly when stratified by sex and year of age; and all data were collected with auscultation while many paediatric practises currently use oscillometric devices.^7^ We sought to examine the feasibility of generating age-, sex- and height-specific BP percentiles using BP measures from PEDSnet (pedsnet.org), a multi-institutional paediatric health system network that currently aggregates electronic health record (EHR) data from more than 8 million children.^8^ EHR data captured from real-world clinic settings directly reflect clinical practise and minimise selection bias. The scope of longitudinal data in PEDSnet offers unprecedented opportunities to study hypertension in children. The objectives of this study were to: 1) identify a study sample of youth 3–17 years of age without evidence of overweight/obesity, chronic diseases, or BP-altering medications, 2) estimate BP distributions for this study sample, and 3) compare these distributions with historically generated BP reference values.

## Methods

### Data source

The study period was 01/01/2009 to 12/31/2021. The PEDSnet database version V.44 was used. Data were extracted in April 2022. Within this time period, there were 8,178,890 children in the database. Member institutions submit data extracts each quarter to the PEDSnet coordinating centre, which evaluates data quality. Participating institutions with data available for this study included Children's Hospital of Philadelphia, Children's Hospital Colorado, Cincinnati Children's Hospital Medical Center, Nationwide Children's Hospital, Nemours Children's Health, Seattle Children's Hospital, Stanford Children's Health, and Lurie Children's Hospital of Chicago.

### Ethics

The study was approved by the Institutional Review Board from Children's Hospital of Philadelphia (IRB 21–018,814). The waiver of authorization was granted since the study analyzed deidentified data from EHR and no informed consent for this study was indicated.

### Study sample

To identify a sample representative of the general paediatric population, we used a set of conservative selection criteria that assured that the children included in this analysis did not have chronic health conditions or treatments that would affect their BP. We included children 3–17 years of age with body mass index (BMI) < 85th percentile during the period of data capture (those with BMI ≥ 85th percentile were excluded) and seen only in outpatient settings, with encounters that included weight, height, and BP measures. Participants were required to have at least two general paediatric care visits (defined as a visit with a general paediatrician, family physician, or non-physician provider—APRN, physician assistant), with at least one such encounter every 18 months if followed for >3 years (approximating recommendations for frequency of well-child visits).^9^ Any method of BP measurement (auscultatory, oscillometric), if recorded, was included.

Blood pressures were only included for outpatient encounters, not inpatient or ED. Patients with chronic conditions at any time during the study period (based on the Paediatric Medical Complexity Algorithm, PMCA,^10^^,^^11^) were excluded. We further restricted the sample by excluding children with exposure to medications that may influence BP, inclusive of antihypertensives, stimulants, and systemic corticosteroids. All codes used for medications are available at https://github.com/PRESERVE-Coordinating-Center/BPZ_Codesets. We excluded children <3 years of age because BP recorded in EHR in this age group was likely obtained from children with preexisting conditions associated with elevated BP. This is consistent with the current recommendation for the general paediatric population to perform annual BP measurements starting at 3 years of age.^6^ Excluding children <3 years of age had little impact on the resulting coefficients in the model since this age group was a small fraction of the sample.

To address the issue of potentially erroneous BP readings that could be spurious, biologically implausible, or indicative of a medical condition that might affect the BP measurement, we used a modification of the approach by the Centres for Disease Control and Prevention to explore potential implausible anthropometric data.^12^ We used BP Z-score (−5/5+) based on preliminary analysis examining extreme categories for the youngest (3 years) and oldest (17 years) children in the sample. For example, for a 3-year-old boy, BP values at Z-scores (−5/5+) were approximately 43/21 and 141/94 and for a 17-year-old boy, BP values at Z-scores (−5/5+) were approximately 65/270 and 173/108, respectively. Similar analyses were performed for each category of age and sex (Supplementary Table S1). There were 362 BP measurements for 344 children outside this Z-score range (0.03% of the sample of measurements, or 0.11% of patients): 178 measurements for systolic BP, and 184 measurements for diastolic BP. Our investigation confirmed that such small samples had no effect on model parameters or predictions.

### Cohort derivation

**Fig. 1:**
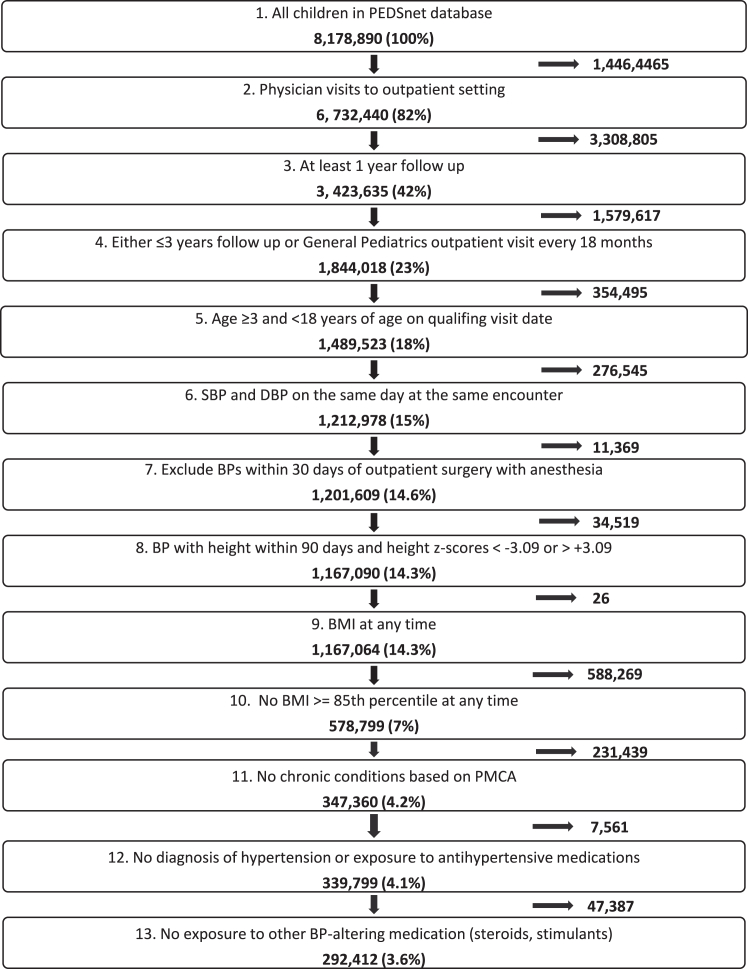
**Cohort sample size derivation.** To identify a sample representing a general paediatric population a 13-step attrition process was implemented.

To examine the attrition procedure for the sample derivation and check for consistency, we used 1% of the PEDSnet population in an exploratory analysis. Across the PEDSnet sites, we observed similar distributions of patient demographic variables and BP values as well as similar changes in cohort retention with each of 13 attrition steps. This was then confirmed with the full sample (Fig. 1, Supplementary Table S2). Because we anticipated that a significant proportion of BP measurements in our sample were obtained by a digital device (which frequently overestimates BP, especially at the first reading), we compared the minimum vs. the average (as recommended by current guidelines^6^) of BP readings per day. Among 1,085,083 BP measurements, in 5.6% the mean BP was greater than the minimum BP with the difference for both systolic and diastolic BP not larger than the standard deviation (σ) in Rosner et al.^5^ Since the data were comparable, we used average BP for the final analysis.

### Statistics

#### Generating Z-scores and BP percentiles

To generate Z-scores and BP percentiles, we first employed a mixed-effects polynomial regression model in which the fixed-effects were first-through-fourth order polynomials of age, sex and height Z-score–the same approach as was utilised in the 2017 AAP CPG^4^^,^^5^ with the following modifications. To be consistent with current guidelines,^6^^,^^13^, ^14^, ^15^, ^16^ we used the average BP measured if more than one BP was available for a given encounter, rather than the first measurement on that day as was done in previous analyses.^4^^,^^5^ Given the large sample size, we divided the study cohort into training (70%) and test (30%) sets, using the training set to create the model and the test set to evaluate the model. To account for change in variability with age and height, mixed-effects models were then created using random effect slopes for age and height Z-score (Supplementary Materials; pp 2–5 for full description of the analyses).

Finally, we pruned the final model of parameters that did not improve model performance. This pruning was based on Akaike Information Criterion (AIC), which is a combination of model likelihood—how likely is it that a model will produce the observed data—and the model complexity. If two models have the same likelihood, the simpler one is preferred. We further pruned the two slopes model of fixed effect terms and confirmed that predictive power was preserved by looking at the correlations (fixed-effect, random-effect and combined) between prediction and actual data in both the full random slopes model and the final model after fixed-effect pruning. This additional pruning of fixed-effects was done to ensure that the model was generalisable by preventing over-fitting.

In addition to mixed-effects polynomial regression to model BP percentiles, we applied quantile regression, the second approach presented by Rosner et al.^5^ In this analysis, the BP value at each percentile was modelled with the same set of predictors as in the mixed-effects polynomial regression model: first-through-fourth-order polynomials of age and of height Z-score, with separate models by BP type (systolic vs. diastolic) and sex (male vs. female). In both the mixed-effects polynomial regression models and quantile regression, we used the fractional year of age, so a patient 6 months past their 10th birthday would have an age of 10.5 as model input. The resulting quantile regression estimates were visually compared to the corresponding mixed-effects model predictions to check for consistency between the two approaches.

#### Comparison to existing normative data

To compare our model performance to that of the model in the 2017 AAP CPG, we assessed intraclass correlation coefficients (ICCs) and compared regressions between the Z-score coefficients in the new model and those from the 2017 AAP CPG.^4^^,^^5^ We also assessed agreement in BP classification between PEDSnet and the 2017 AAP CPG by calculating Cohen's kappa for ordinal data with a squared-weighting which increases penalty for further separation between categories, using the Z-score thresholds from Banker et al.^17^: 10th percentile, 90th percentile, 95th percentile, and 95th percentile +12 mmHg. As suggested by Cohen, values ≤ 0 would indicate no agreement, 0.01–0.20 slight, 0.21–0.40 fair, 0.41–0.60 moderate, 0.61–0.80 substantial, and 0.81–1.00 excellent agreement.^18^

To check whether differences between models were due to systematic trends over time, we performed a regression between Z-scores and calendar year. We also constructed empirical cumulative distribution function curves to examine whether model predictions were consistent among males and females, different race/ethnic groups, and across different health systems.

Analyses were performed with R software, version 4.3.1 (R Core Team 2023; https://www.R-project.org).

### Role of funders

The funding sources did not have any role in study design, data collection, data analyses, interpretation, or writing of the report.

## Results

### Study population characteristics

Data from 292,412 children (1,085,083 BP measurements) who met inclusion criteria during the period 01/01/2009–08/31/2021 contributed to this study. The characteristics for the PEDSnet population sample were compared with those from the 2017 AAP CPG, Table 1. The 2017 AAP CPG population had a higher percentage of Black or African American children, while PEDSnet had a higher percentage of Asian children. Demographic characteristics at each contributing PEDSnet centre are shown in Supplementary Table S3. The number of patients contributing to each year of age category varied from approximately 45,000–75,000 children (Supplementary Table S4). Our dataset included more measurements per child during the study period: mean of 3.7 for both systolic and diastolic BP vs. means of 1.2 for systolic and 1.3 for diastolic BP measurements in the 2017 AAP CPG. Approximately 27% of our cohort had only one encounter, 21% - 2, 14% - 3, 10% - 4, 7% - 5 and 20% had ≥6 encounters. Among those getting multiple encounters, the spans (max value minus min value) for age were median 2.2 years (IQR 1.2–4.4) and for height Z score were 0.4 (IQR 0.2–0.7).

**Table 1:** Comparison of demographic characteristics of children contributing to PEDSnet and 2017 AAP CPG samples.

	PEDSnet	2017 AAP CPG
Age range, years	3–17	1–17
Sex		
Boys	137,598 (47%)	27,627 (51%)
Girls	154,814 (53%)	24,316 (49%)
Race/Ethnicity		
Non-Hispanic Asian/Pacific Islander	19,725 (6.7%)	1505 (3%)
Non-Hispanic Black/African-American	55,252 (18.9%)	14,471 (29%)
Hispanic	20,484 (10.4%)	4429 (9%)
Multiple	6844 (2.3%)	–
Other/Unknowns^a^	38,814 (13.3%)	1421 (3%)
Non-Hispanic White	141,293 (48.3%)	27,627 (55%)
Sample size		
Children with systolic blood pressure	292,412	49,967
Encounters with systolic blood pressure	1,085,083	65,439
Children with diastolic blood pressure	292,412	36,914
Encounters with diastolic blood pressure	1,085,083	45,017

a Includes small groups such as Native Americans as well as patients for whom no information is provided.

### Age-, sex- and height-specific BP Z-scores

Direct comparison with AAP CPG model is presented in Table 2. It displays the results of the mixed-effects polynomial regression model with random intercept only in which the fixed-effects were first-through-fourth order polynomials of age, sex, and height Z-score.

**Table 2:** PEDSnet mixed-effects polynomial blood pressure regression model (random intercept, for comparison with 2017 AAP CPG).

	Systolic blood pressure				Diastolic blood pressure			
	Boys (n = 137,598)		Girls (n = 154,814)		Boys (n = 137,598)		Girls (n = 154,814)	
	Coefficient	95% CI	Coefficient	95% CI	Coefficient	95% CI	Coefficient	95% CI
Intercept	103.88965	103.81320, 103.96610	104.04188	103.96716, 104.11660	62.26646	62.20895, 62.32397	62.46709	62.41456, 62.51961
Age–10	1.71846	1.69913, 1.73780	1.65051	1.63289, 1.66813	0.74699	0.73212, 0.76185	0.79915	0.78553, 0.81278
(Age–10)^2^	0.11811	0.11183, 0.12439	−0.00118	−0.00679, 0.00443	−0.01614	−0.02103, −0.01125	−0.00742	−0.01181, −0.00303
(Age–10)^3^	−0.00064	−0.00113, −0.00015^a^	−0.00691	−0.00735, −0.00647	−0.00146	−0.00184, −0.00108	−0.00249	−0.00283, −0.00214
(Age–10)^4^	−0.00144	−0.00156, −0.00132	−0.00031	−0.00042, −0.00021	0.00042	0.00033, 0.00051	0.00014	0.00005, 0.00022
Height-Z	1.25749	1.18791, 1.32708	0.98468	0.94389, 1.02547	0.36553	0.33320, 0.39785	0.38153	0.35111, 0.41194
Height-Z^2^			0.06251	0.03456, 0.09047				
Height-Z^3^	−0.02279	−0.04086, −0.00472^a^						
Height-Z^4^	0.01146	0.00658, 0.01633			0.00694	0.00329, 0.01060		
σ	9.644		9.514		7.312		7.261	
ICC	0.34		0.34		0.27		0.28	
Fixed-effect R^2^								
*Test Set*	0.401		0.318		0.133		0.140	
*Training Set*	0.399		0.316		0.137		0.141	

Data for all variables are presented with similar decimal points as in 2017 AAP CPG^4^^,^^5^ for direct comparison.

CI, Confidence intervals; ICC, Intraclass correlation.

An example to calculate BP percentiles using parameters from the table for a 12-year-old boy and Height at the 90th percentile (Height Z = 1.28), the age-10 is 2 and so the expected Systolic BP is 103.88965 + 1.71846 (2) + 0.11811 (2^2^) + 0.00249 (2^3^) − 0.00144 (2^4^) + 1.25749 (1.28) − 0.02279 (1.28^3^) + 0.01146 (1.28^4^) = 109.39 mmHg. If the child's actual BP were 120 mmHg, then the Z-score would be (120–109.39)/9.644 = 1.100. From the Gaussian distribution, BP percentile associated with that Z score is 86.4th.

All parameters significant to p < 0.0001.

a p < 0.01.

The coefficients for model in PEDSnet (intercepts approximately 104 for systolic BP and 62 for diastolic BP for both males and females) were comparable to those presented in the 2017 AAP CPG (approximately 102 for systolic BP and 61 for diastolic BP).^4^^,^^5^ The ICC, the proportion of the overall variance attributable to patient-level effects in a mixed-effects model indicates that approximately one third of the variance in BP measurements can be attributed to variations among children, and the rest attributable to variations between measurements taken on different days (e.g., stress level, time of the day or seasonal variation). Standard deviation (SD) in PEDSnet was smaller (approximately 9.5 mmHg for systolic BP and 7.5 mmHg for diastolic BP) compared to approximately 10 mmHg for systolic BP and 11 mmHg for diastolic BP in 2017 AAP CPG.

**Fig. 2:**
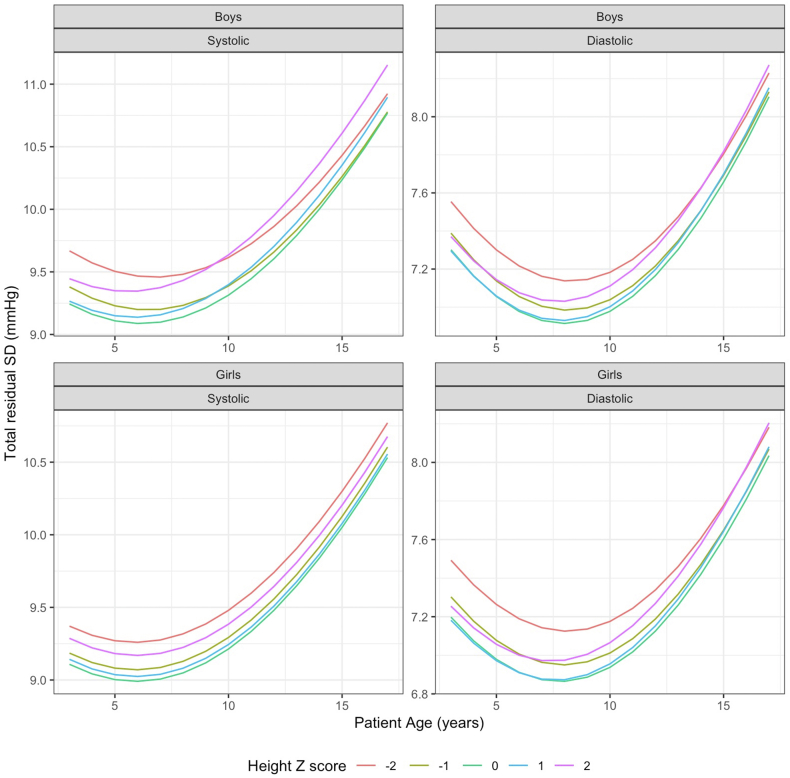
**Dependence of overall standard deviation (for calculating Z score) on age and height Z.** The parameters describing this variation are presented in Table 3 and the formulae are in Supplementary Material. Note that despite the variation, the overall number stays close to the values in Table 2.

To account for change in variability with age and height, mixed-effects models were updated by using random effect slopes for age and height Z-score, Table 3. In this model, ICC and SD vary with age and height, although the numbers were comparable, Fig. 2. Adding random slopes improved model likelihood with significant (p < 0.001; likelihood ratio test with optimised log-likelihood) drop in AIC for both analyses: from no slope to age slope, and from age slope to both slopes. This model was then used to generate BP percentiles and for comparison analysis. Technical details for the implementation of this model and sample R code are presented in the Supplementary Materials, pp 2–5.

**Table 3:** PEDSnet mixed-effects polynomial blood pressure regression model with random effect slopes for age and height Z-score (Final model).

	Systolic blood pressure				Diastolic blood pressure			
	Boys (n = 137,598)		Girls (n = 154,814)		Boys (n = 137,598)		Girls (n = 154,814)	
	Coefficient	95% CI	Coefficient	95% CI	Coefficient	95% CI	Coefficient	95% CI
Intercept	103.81317	103.73846, 103.88787	103.97061	103.91350, 104.02772	62.22508	62.16977, 62.28038	62.41751	62.36578, 62.46923
Age–10	1.73361	1.71389, 1.75333	1.64756	1.62972, 1.66540	0.67985	0.66474, 0.69495	0.72471	0.71084, 0.73858
(Age–10)^2^	0.12287	0.11652, 0.12921			−0.01321	−0.01813, −0.00830	−0.00411	−0.00853, 0.00031^b^
(Age–10)^3^	−0.00068	−0.00117, −0.00019^a^	−0.00673	−0.00716, −0.00630	−0.00033	−0.00072, 0.00005^b^	−0.00126	−0.00160, −0.00091
(Age–10)^4^	−0.00149	−0.00161, −0.00137	−0.00030	−0.00161, −0.00137	0.00039	0.00029, 0.00048	0.00010	0.00002, 0.00018^a^
Height-Z	1.24648	1.17639, 1.31658	0.98278	0.94132, 1.02423	0.36050	0.32745, 0.39355	0.37092	0.33973, 0.40211
Height-Z^2^			0.07001	0.04116, 0.09887				
Height-Z^3^	−0.02799	−0.04691, −0.00906^a^						
Height-Z^4^	0.01454	0.00933, 0.01975			0.00720	0.00334, 0.01106^a^	0.00410	0.00035, 0.00786^b^
σ _Intercept_	5.03383		5.02626		3.28489		3.34873	
ρ _Intercept, age10_	0.34877		0.3473		0.20928		0.22796	
ρ _Intercept, height-Z_	−0.0007		−0.04389		−0.05694		−0.07968	
σ _Age10_	0.53342		0.49579		0.4688		0.45282	
ρ _Age10, height-Z_	0.12559		−0.00832		0.08343		0.09753	
σ _Height-Z_	1.21332		1.00908		0.77311		0.80022	
σ _Observation_	7.76685		7.65618		6.12374		6.04074	
Fixed-effect R^2^								
*Test Set*	0.401		0.318		0.133		0.140	
*Training Set*	0.399		0.316		0.137		0.141	

Data for all variables are presented with similar decimal points as in 2017 AAP CPG^4^^,^^5^ for direct comparison.

Because of the more complicated mathematics, the detailed example is in the Supplementary Materials, pp 2–5. To sum up, using the same scenario as in the 4th Report and Table 2, the conditional mean systolic BP for a 12-year-old boy (age 10 = 2) and Height at the 90th percentile (Height Z = 1.28) is: 103.81317 + 1.73361(*2*) + 0.12287(*2*^*2*^)—0.00068(*2*^*3*^) − 0.00149(*2*^*4*^) + 1.24648(*1.28*) + 0(*1.28*^*2*^) + 0.02799(*1.28*^*3*^) + 0.01454(*1.28*^*4*^) = **109.32 mmHg**. In the AAP CPG, the corresponding calculation predicted 109.46 mmHg. The SD is 9.66368 (see Supplementary Materials) so if the child's actual BP were 120 mmHg, then the Z-score would be (120–109.46)/9.66368 = 1.091, which corresponds to the 86.2nd percentile.

CI, confidence intervals; σ, Standard deviation; ρ, Correlation.

σ _Intercept_ is the square root of the variance associated with the random-effect intercept term, corresponding to a per-person variation in mean BP.

σ _age10_ is the square root of the variance associated with the random slope term for age 10.

σ _height-Z_ is the square root of the variance associated with the random slope term for height Z score.

ρ _Intercept, age10_ is the correlation between the random effect parameters for intercept and the slope for age 10. The covariance of the two terms is equal to ρ_Intercept, age10_(σ_Intercept_) (σ_age10_).

ρ _Intercept, height-Z_ is the correlation between the random effect parameters for intercept and the slope for height-Z. The covariance of the two terms is equal to ρ_Intercept, height-Z_ (σ_Intercept_) (σ_height-Z_).

ρ _age10, height-Z_ is the correlation between the random effect parameters for intercept and the slope for height-Z. The covariance of the two terms is equal to ρ_age10,__height-Z_ (σ_age10_) (σ_height-Z_).

All parameters significant to p < 0.0001.

a p < 0.01.

b ^+^p < 0.05.

### Generalisability of model

Building the model on a 70% training set and comparing the predictors for that set to a 30% of test set showed that the R-squared values, the intercepts, and slopes were consistent between the test set and training set (Supplementary Table S5). When we compared the original model and model based on “pruned” data (models with and without exclusion of potentially erroneous data), the results were not different: the difference between Z scores (Z pruned model—Z existing model) had a range of −0.088 to +0.021, with a mean of −0.001 ± 0.003. Predictions were also consistent among males and females, different race/ethnic groups, and across different health systems (Supplementary Figure S1a-c).

### Comparison of new and 2017 AAP CPG model

**Fig. 3:**
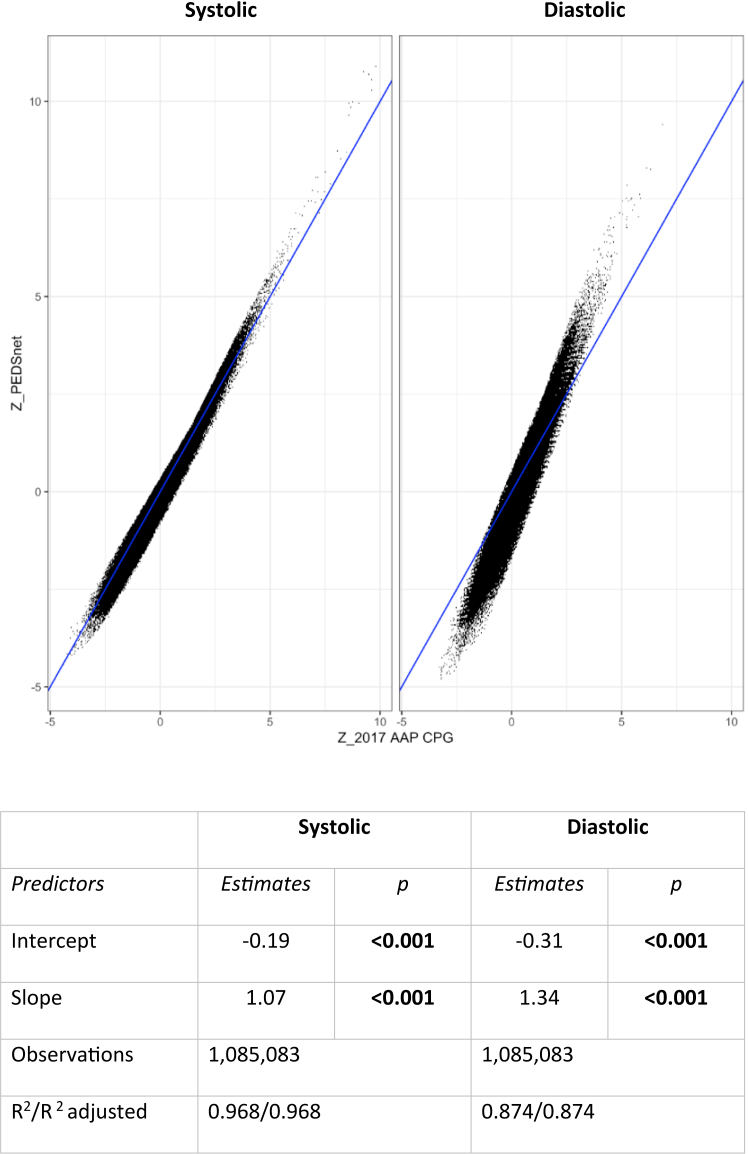
**Blood pressure z-scores comparison: PEDSnet vs. 2017 AAP CPG.** The blue line is defined as y = x. Linear regression model is Z (new) = Intercept + slope ∗Z (2017 AAP CPG). p-values based on Wald tests. The negative intercepts suggest that the average new Z is less than the average 2017 AAP CPG Z that is our data may show a slight tendency towards higher BP of ∼0.1 SD. An R-squared of 0.971 for systolic BP and 0.885 for diastolic BP indicates strong agreement between the Z-scores from 2017 AAP CPG and our new model.

The results from PEDSnet and 2017 AAP CPG models were strongly correlated with an R-squared of 0.968 for systolic BP and 0.873 for diastolic BP (Fig. 3). Cohen's Kappa (Supplementary Table S6) to assess the agreement between BP categories in the models was 0.90 for systolic BP, 0.64 for diastolic BP, and 0.79 overall indicating excellent agreement between PEDSnet and 2017 AAP CPG data for systolic BP and substantial agreement for diastolic BP for clinically relevant elevated BP categories (≥90th percentile). These results were consistent when we limited the patient sample to ages 3–12 years (0.89 Systolic, 0.67 Diastolic, 0.79 overall), as classification for BP categories for ≥13-year-old adolescents is based on absolute BP measurements (U.S. adult criteria).

A linear regression of BP Z-scores from the PEDSnet model vs. time to assess the effects of calendar year on model coefficients showed that systolic BP Z-scores decreased at a rate of 0.009 units on average per calendar year, while diastolic BP Z-scores increased at a rate of 0.030 units per calendar year.

**Fig. 4:**
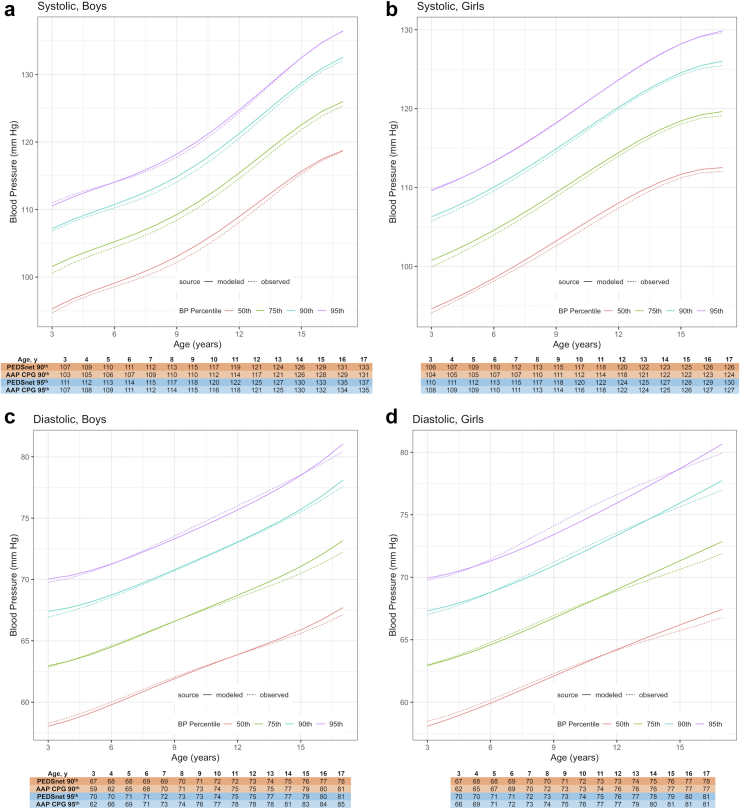
**a–d: Systolic and diastolic BP 50th, 75th, 90th, and 95th percentiles by age and height 50th percentile for boys and girls**. The solid lines are the percentiles from mixed-effects polynomial regression. The dashed lines are BP percentiles from quantile regression. The results from both methods were similar. Tables show comparison of PEDSnet and 2017 AAP CPG BP values at 90th and 95th percentiles for boys and girls in 50th height percentile. Systolic BP values are higher in PEDSnet than 2017 AAP CPG across all age categories, mostly in younger children. Diastolic BP values are higher in PEDSnet in young children (3–5-year-old) but lower in older children than in 2017 AAP CPG.

Fig. 4a–d shows BP percentile values according to age and sex for children in the 50th percentile for height (based on the model with random effect slopes). Compared to 2017 AAP CPG values, PEDSnet systolic BP percentile values were slightly higher (e.g., 1–4 mmHg for 90th and 95th percentiles) across different age, sex, and height categories, with greater differences seen in younger children. While PEDSnet diastolic BP percentile values were also higher in younger children, starting with age 7 in both males and females, diastolic BP percentile values were 1–2 mmHg lower than 2017 AAP CPG values. In contrast to the 2017 AAP CPG tables in which diastolic BP percentiles were higher for males than females, we did not observe sex differences for diastolic BP percentile values across different age and height percentiles. Similar BP increases with age were seen across different height percentiles (Supplementary Figure S2a–d). Full tables for these values are provided in Supplementary Table S7 and Supplementary Table S8.

To assess the impact of PEDSnet derived percentiles on BP classification, we used our dataset to determine the prevalence of BP ≥ 95th percentile among children 3–12 years of age. For systolic BP, 11.2% of children who had BP ≥ 95th percentile using 2017 AAP CPG model had their BP classified as < 95th percentile using PEDSnet model. For diastolic BP, in the 3–5-year age category, 8.5% were reclassified as having BP < 95th percentile; among 6–12-year-old none were reclassified as having BP ≥ 95th percentile using PEDSnet model.

## Discussion

This study illustrates the feasibility of using “real-world” EHR data to develop empirical distributions for a clinical variable such as BP. We used data from more than one million BP measurements among almost 300,000 normal-weight children. The largest datasets prior to our study compiled BP data obtained from approximately 50,000 U.S. children included in the 2017 AAP CPG^6^ and from approximately 53,000 children who contributed to the development of international BP percentiles from seven countries.^19^ Compared to these research-based BP references, our estimated distributions are based on contemporary BP data accumulated over the last decade across major paediatric health centres representative of the diverse U.S. paediatric population and reflective of current clinical practise for BP measurement.

Our results indicate that the PEDSnet models and derived BP percentiles align closely with the 2017 AAP CPG model and reference values as shown by similarity in BP coefficients, ICC between models and strong Cohen's Kappa agreement, especially for systolic BP. The new mixed model with random effect slope for age and height Z-score improved relationships between percentiles from quantile regression and percentiles estimated from the mixed-effects model. Even though adding random slopes increased model complexity, it drastically improved model likelihood, and addressed the concern raised by Rosner et al.^5^ that the random-intercept model used previously was based on a constant SD across the population and did not allow for variation over different ages and heights.

PEDSnet systolic BP values were slightly higher; diastolic BP values were also higher in young children but slightly lower than in the AAP CPG generated tables in older children. The primary explanation for this difference is that a significant proportion of our cohort likely had their BP measured using oscillometric devices. A recent study to assess paediatrician adherence to the 2017 AAP CPG found that 54% of practises used an oscillometric device for initial BP screening, 39% measured BP manually, and the remainder used a combination of techniques.^7^ Thus, we assume that BP data in our cohort represents a combination of both manual and oscillometric measures reflecting current practise, while previously published references were based on BP measured by auscultation only. Both systolic and diastolic BP percentile values calculated in our study were similar to oscillometric BP normative standards for Swedish children^20^ also supporting the case that a substantial number of children in PEDSnet had BP measured by a digital device. Use of oscillometric devices can also explain slightly lower diastolic BP in our study than in the 2017 AAP CPG as was shown in a cross-over, population-based study from Iceland^21^ and in a study from Germany (in which diastolic BP percentiles in younger adolescents were similar to our study).^22^ In older adolescents, German children showed similar to AAP CPG diastolic BP percentile values. We considered adding a method of BP measurement (manual or oscillometric) to refine the model, but these data were not reliably reported in the EHR. We also observed that despite being shorter, diastolic BP percentile values in females were similar to those of males for a given age and height percentile. This indicates that height may be less important in determining diastolic BP compared to systolic BP. Alternatively, these results might highlight a limitation in determining true diastolic BP percentiles using data from EHR.

As recommended by all major guidelines,^6^^,^^13^, ^14^, ^15^, ^16^^,^^23^ we included the average of measured BP readings on a given day to generate BP percentiles rather than the first measurement as was done by previous analyses upon which the 2017 AAP CPG tables are based.^4^^,^^5^ Recommendations to use average BP apply to both manual and oscillometric BP measurements, but are especially relevant when digital devices are used. A study using a digital monitor in adults estimated that 35% of participants with stage 2 hypertension based on their initial reading had BP < 140/90 mmHg when the average of 3 measurements was used; in participants with elevated or stage 1 hypertension on their first BP measurement, 24% had normal BP based on the average of their second and third measurements.^24^ In a large paediatric study, if the average of two oscillometric BP readings was used during the visit, an initial BP ≥ 95th percentile was not confirmed in 52% of children.^25^ Finally, less variation among patients and less day-to-day variation within patients in the PEDSnet analysis might indicate that some of the differences between PEDSnet and 2017 AAP CPG BP values might also be due to differences in the study population used in the analysis (e.g., differences in sample size or temporal trends in paediatric population health over the last few decades).

The use of EHR data in research has greatly accelerated over the last decade in areas ranging from informing clinical trial design to comparative effectiveness, assessing drug efficacy, standardising care and care delivery, and others.^26^ There are known limitations in the use of EHR data, mostly related to data quality and potential bias. In our study, we could not control for BP measurement technique since data on BP cuff size, arm circumference, resting time, and equipment used (manual vs. oscillometric) to assess BP were not uniformly recorded in the EHR. While we tried to restrict our sample to a general paediatric population and excluded children with chronic conditions, some children might have had acute illnesses that could affect the results of the study. However, our study has significant strengths including a diverse and contemporary cohort with much larger sample size than previous data used to generate paediatric BP normative values. Importantly, our data are reflective of current clinical practise in terms of how BP is measured and classified by paediatric providers for decision-making on management of a child with elevated BP.

Application of real-world developed BP percentiles may have important implications for BP management in children, including follow up, evaluation, referral to subspecialist and treatment. For example, while our results would not affect the diagnosis of elevated BP or hypertension in children ≥13 years of age for whom current AAP CPG BP classification is based on fixed adult criteria, some younger children (<7 years of age) who were classified as having high systolic or diastolic BP based on 2017 AAP CPG BP percentiles would not be diagnosed with elevated BP or hypertension based on our estimated percentile values. A small number of children aged 7–12 years would be reclassified as having diastolic BP in the hypertensive range because PEDSnet diastolic BP percentiles in this age group were generally lower.

Our methodology could readily be applied to other EHR data sources to confirm distributions, such as Comparative Effectiveness Research through Collaborative Electronic Reporting (CER^2^) and the Child Health Improvement through Longitudinal Data (CHILD) registry. Generating empirical distributions of BP values using large samples from the EHR is feasible and cost-effective. The data could be efficiently updated on a periodic basis (e.g., once a decade) to reflect changes in US demographics or in clinical practise of BP management. Future studies to assess the effect of EHR-derived BP percentiles on the evaluation of hypertension and hypertension-related target organ damage need to be conducted before these data could be adopted into clinical practise.

## Contributors

All authors read and approved the final version of the manuscript.

**MMM:** Data collection, design of the study, analysis, draught of the manuscript; **MM**: Formal statistical analysis (main statistician for this study), data verification, writing, reviewing and editing; **MRD**: Data collection and verification, design of the study, analysis, writing, reviewing and editing; **JTF**: Data collection, reviewing and editing; **JS**: Data analysis, project manager; **BPD**: Data collection, analysis, reviewing and editing; **HRP**: Data collection, analysis, reviewing and editing; **DJC**: Data collection, analysis, reviewing and editing; **KD**: Data curation and verification; **YC**: Formal statistical analysis (validation of the results), reviewing and editing; **CG**: Data collection, reviewing and editing; **ML**: Data collection, reviewing and editing; **PSV**: Data collection, reviewing and editing; **CBF**: Data collection and verification, design of the study, analysis, writing, reviewing and editing.

## Data sharing statement

All codes used for medications are available at https://github.com/PRESERVE-Coordinating-Center/BPZ_Codesets. An R function (code sharing) that implements this model is provided in Supplementary Materials. The data that support the findings of this study are available from the PRESERVE study principal investigators upon reasonable request.

## Declaration of interests

The authors declare that they have no known competing financial interests or personal relationships that could have appeared to influence the work reported in this paper.
