# Supplementary material for: Derivation of paediatric blood pressure percentiles from electronic health records
Source: eBioMedicine. 2023 Nov 19;98:104885. doi: 10.1016/j.ebiom.2023.104885 (PMC10679476; doi:10.1016/j.ebiom.2023.104885)
Supplement: Supplementary Material [file mmc1.docx]

**Supplementary Material**

1. **Statistical analysis; pp 2-5**
   1. **Implementation of the mixed-effects polynomial regression model (pp 2-3)**
   2. **R code (pp 4-5)**
2. **Supplementary Figures; pp 6-7**

**Supplementary Figure S1a-c. Variation of systolic and diastolic BP Z-score by a) Sex, b) Race/ethnicity, and c) Health systems.** Dash line indicates z-score 1.65. Empical cumulative density functions calculated as % of measurements at or above a given level

**Supplementary Figure S2a-d: Systolic and diastolic BP at the 90^th^ percentile by age and different height percentiles for boys and girls.** BP increase with age was similar for different height percentiles.

1. **Supplementary Tables; pp 8-22**

**Supplementary Table S1. Sample size derivation by site.**

**Supplementary Table S2: BP (mmHg) values for Z-scores (-5/5+) by age, sex, and height 50^th^ percentile.**

**Supplementary Table S3. Demographic characteristics by health system.**

**Supplementary Table S4. The number of children contributed to analysis by age and health system.**

**Supplementary S5. Regressions between observation and model prediction for the test and training sets.** Intercept and slope are consistent within the limits of the standard error of the estimate.

**Supplementary Table S6. Comparisons of BP classifications between new model and 2017 AAP CPG with Cohen’s Kappa category counts**

**Supplementary Table S7. BP Levels for Boys by Age and Height Percentile**

**Supplementary Table S8. BP Levels for Girls by Age and Height Percentile**

1. **References; pp 23**

**Statistical Analyses**

Analyses were performed with R software, version 4.3.1. R Core Team (2023). R: A language and environment for statistical computing. R Foundation for Statistical Computing, Vienna, Austria. [https://www.R-project.org](https://www.R-project.org/). The package within R used for the mixed-effects model was lme4, version 1.1.(8) R package version 5.94 was used for quantile regression model (Koenker R (2022). Quantile Regression. <https://CRAN.R-project.org/package=quantreg>.

- 1. **Implementation of the mixed-effects polynomial regression model**

The initial work by Rosner et al(7) and in the Fourth Report(1) used a random-intercept model which allows for the fact that each person might have a different average BP. Rosner et al(7) point out that a potential limitation in this approach is that the estimate of total variance (the combination of patient-level differences and measurement variation) is assumed constant over the lifespan of the patient.

As described in the main text, we found that adding “random slope” terms that allowed the relationship between BP and either age or height percentile to vary among patients improved model fits substantially. Using the lme4 package in R, the resulting mixed-effects polynomial regression model was implemented with calls of the structure:

lmer(bp~age10 + I(age10^2) + I(age10^3) + I(age10^4) + heightZ + I(heightZ^2) +I(heightZ^3) + I(heightZ^4) + (age10 + heightZ|ID)

where age10 was the age minus ten years (for consistency with (1) and (7)) and height Z was the Z-score for height based on CDC tables.

To accurately compare models using AIC, the REML=FALSE option was used as random effects or fixed effects were changed. When a final model was determined, the model was recalculated using REML=TRUE to produce unbiased estimates of variance and covariance parameters.

The resulting fixed-effect and random-effect parameters for the final random-slopes model are presented in Table 3, while a comparable model using only random intercept is presented in Table 2. We recommend using the Table 3 coefficients because of the better performance and provide R code for implementing the model. An example to implement the model is shown below. This example is taken from the Fourth Report (1), systolic BP of 120 mmHg a 12-year-old boy with height in the 90^th^ percentile, so height Z score is 1.28 and age10 is 2.0.

As in the Fourth Report, the Z score is estimated as the difference (BP_observed_ – BP_predicted_)/SD_total_, where BP_observed_ is the actual measurement and BP_predicted_ is the prediction from fixed effects, and SD_total_ is based on the combined variance from the measurement-level variation and the patient-level variation. In (1) and (7) SD total is a constant, but not for the new model.

As before, the conditional mean for the BP is calculated from the intercept and the fixed effects as intercept plus the sum of the polynomial terms for age minus 10 years and the polynomial terms for height Z score, or

BP_predicted_ =

(Intercept) + A_age10_ * *age10* + A_age10_^2^ * *age10^2^* + A_age10_^3^ * *age10^3^* + A_age10_^4^ * *age10^4^* + B_heightZ_ * *heightZ* + B_heightZ_ ^2^ * *heightZ ^2^* + B_heightZ_ ^3^ * *heightZ ^3^* + B_heightZ_ ^4^ * *heightZ ^4^*

For our example, the expected SBP is:

103.81317+ 1.73361(*2*) + 0.12287(*22*) - 0.00068(*23*) - 0.00149(*24*) + 1.24648(*1.28*) + 0(*1.28^2^)* + 0.02799(*1.283*) + 0.01454(*1.284*) **= 109.32 mmHg.** In the Fourth report, the corresponding calculation predicted 109.46 mmHg.

The three random effects para meters are assumed to come from a three-variable Gaussian distribution, defined by the variance of each term, the correlation between them, and for the slope terms, the specific values of the parameters.

Using the parameters returned by the linear mixed model (Table 2), this random-effects variance is estimated as the sum:

σ^2^_random_ = σ^2^_Intercept_ + (σ_age10_)^2^ (*age10*)^2^ + (σ_height-Z_)^2^ (*height-Z*)^2^ +

2(ρ_Intercept, age10_)( σ_Intercept_)( σ_age10_)(*age10*) +

2(ρ_Intercept, height-Z_))( σ_Intercept_ ( σ_height-Z_))( *height-Z*) +

2(ρ_age10, height-Z_))( σ_age10_)( σ_height-Z_)) (*age10*) ( *height-Z*)

The resulting total SD (SIG in the 4^th^ report model) is the square root of the sum of the observation level variance and the random-effects variance, σ^2^_observation_ + σ^2^_random_. In our example, the corresponding random-effects variance is

σ^2^_random_ = 5.03383^2^ + 0.53342^2^ (*2^2^*) + 1.21332^2^(*1.28^2*) +

2(0.34877)( 5.03383)(0.53342)(*2*) +

2(0.00070)(5.03383)(1.21332)(*1.28*) +

2(0.12559)(0.53342)(1.21332)(*2*)(*1.28*) =

**33.06266 (mmHg^2).**

The fixed-effects variance is (7.76685^2^) so the total SD is sqrt(33.06266+7.76685^2^) = **9.66368,** compared to 10.7128 in the corresponding 4^th^ report parameter.

If the measured systolic BP were 120 mmHg, then the Z-score would be (120-109.46)/9.66368 = 1.091, which corresponds to the 86.2^nd^ percentile.  In the 4^th^ report example, the corresponding predicted blood pressure was 109.46 mmHg, and the associated Z-score was 0.984, or the 83.7^th^ percentile.

Note that in these calculations, age is based on elapsed time from date of birth, so exact years. In tables from Flynn et al (6) paper, the ages assume midpoint of the year, generated by adding a 6.5 month offset (<https://sites.google.com/a/channing.harvard.edu/bernardrosner/pediatric-blood-press/childhood-blood-pressure/instructions>). Where we provide results by year of age in this paper, the same convention is followed. Such calculations were used in Figures 4A-D, Appendix Figure E2A-D, Tables E7 and E8.

- 1. **An R function** that implements this model (with an option for the 6.5-month adjustment) is:

***bp_score_with_slopes <- function(id, bp_observed, type, sex, htZ, age_yrs, adjust_years = FALSE) {***

***require(dplyr)***

***require(tibble)***

***require(magrittr)***

***params <- tribble(***

***~sex, ~type, ~Intercept, ~`I(age10)`, ~`I(age10^2)`, ~`I(age10^3)`, ~`I(age10^4)`, ~`I(htZ)`, ~`I(htZ^2)`, ~`I(htZ^3)`, ~`I(htZ^4)`, ~sd__Observation, ~sd__Intercept, ~sd__age10, ~sd__htZ, ~cor__Intercept.age10, ~cor__Intercept.htZ, ~cor__age10.htZ,***

***"Girls", "Systolic", 103.970610975827, 1.64755776974921, 0, -0.00672854936027804, -0.000304445721417763, 0.982775921614132, 0.0700148212162216, 0, 0, 7.65618196100167, 5.02626404695056, 0.495786808711336, 1.00907755650047, 0.347301151089185, -0.0438939634213447, -0.00831989703701446,***

***"Boys", "Systolic", 103.813165414558, 1.73361093417982, 0.122865372703926, -0.000680228403270637, -0.00149262751588901, 1.24648445144301, 0, -0.02798685938599, 0.0145394234636759, 7.7668492380853, 5.03382723203088, 0.533416731134191, 1.21332496513462, 0.348769779023855, -0.000699532919060102, 0.125593847935152,***

***"Girls", "Diastolic", 62.4175056948638, 0.72470805379935, -0.00411259130419403, -0.00125858316711285, 0.00010093708937658, 0.370921898995395, 0, 0, 0.00410451358945263, 6.04074129850983, 3.34872609310043, 0.452819518316222, 0.800219885567322, 0.227962461614995, -0.0796808572848491, 0.097529826610063,***

***"Boys", "Diastolic", 62.2250773400853, 0.679848858931832, -0.0132139847486287, -0.000331687777961079, 0.00038557852325833, 0.360497215264303, 0, 0, 0.0071988459815278, 6.1237444560795, 3.28489191426022, 0.468796616085828, 0.773108791464125, 0.209282231809696, -0.0569401952308633, 0.0834302520876097)***

***tmp <-***

***tibble(id = id, sex = sex, bp_observed = bp_observed, type = type, htZ = htZ, age_yrs = age_yrs) %>%***

***mutate(age10=case_when(adjust_years==TRUE~age_yrs-10.0+6.5/12.0, TRUE~age_yrs-10.0))***

***rslt <-***

***tmp %>%***

***inner_join(params) %>%***

***mutate(***

***bp_predicted =***

***Intercept +***

***`I(age10)` * age10 + `I(age10^2)` * age10^2 + `I(age10^3)` * age10^3 + `I(age10^4)` * age10^4 + `I(htZ)` * htZ + `I(htZ^2)` * htZ^2 + `I(htZ^3)` * htZ^3 + `I(htZ^4)` * htZ^4 ) %>%***

***mutate(***

***random_var =***

***sd__Intercept^2 +***

***(age10 * sd__age10)^2 +***

***(htZ * sd__htZ)^2 +***

***2 * cor__Intercept.age10 * sd__Intercept * sd__age10 * age10 +***

***2 * cor__Intercept.htZ * sd__Intercept * sd__htZ * htZ +***

***2 * cor__age10.htZ * sd__htZ * sd__age10 * htZ * age10) %>%***

***mutate(total_sd = sqrt(random_var + sd__Observation^2)) %>%***

***mutate(new_Z_score = (bp_observed - bp_predicted) / total_sd) %>%***

***mutate(fixed_sd = sd__Observation, random_sd = sqrt(random_var))***

***return(rslt)***

***}***

**Supplementary Figure S1a-c. Variation of systolic and diastolic BP Z-score by a) Sex, b) Race/ethnicity, and c) Health systems.** Dash line indicates z-score 1.65. Empical cumulative density functions calculated as % of measurements at or above a given level

**b. Race/ethnicity**

**a. Sex categories**


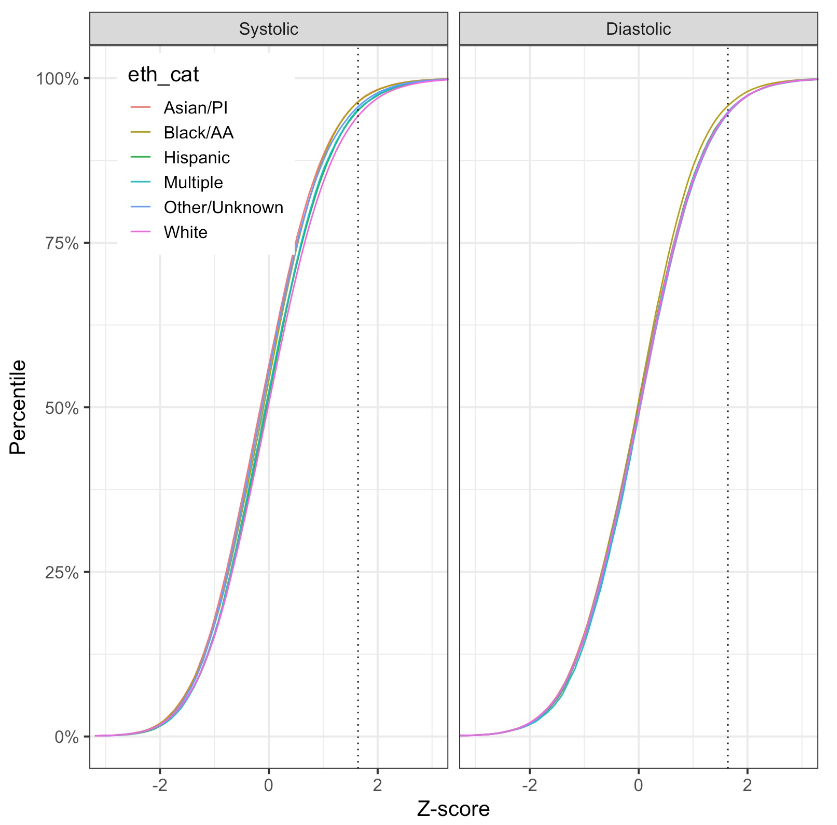

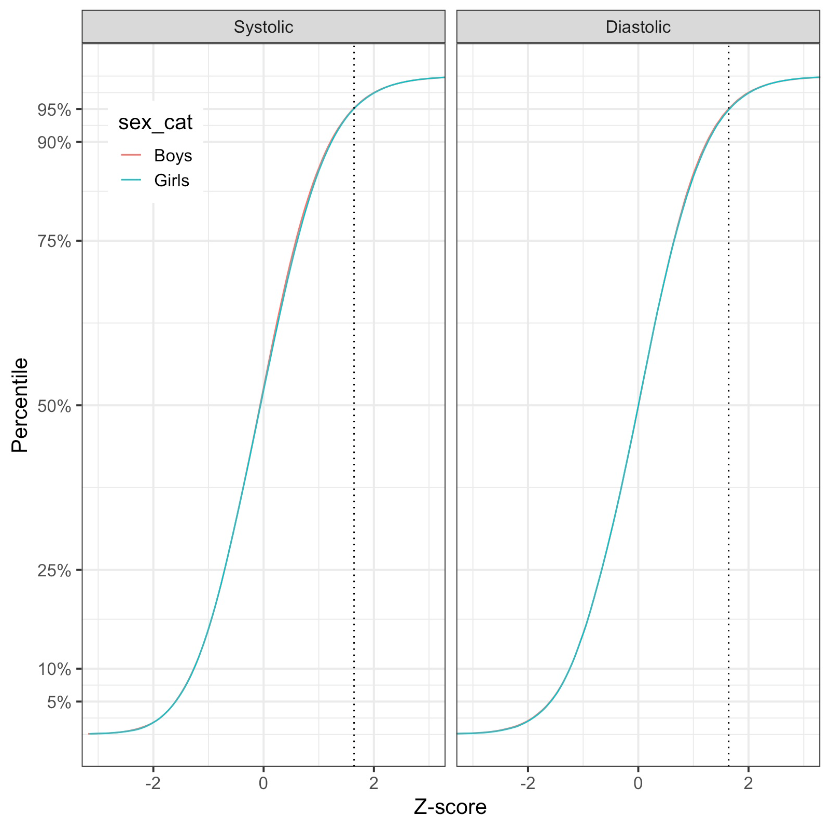


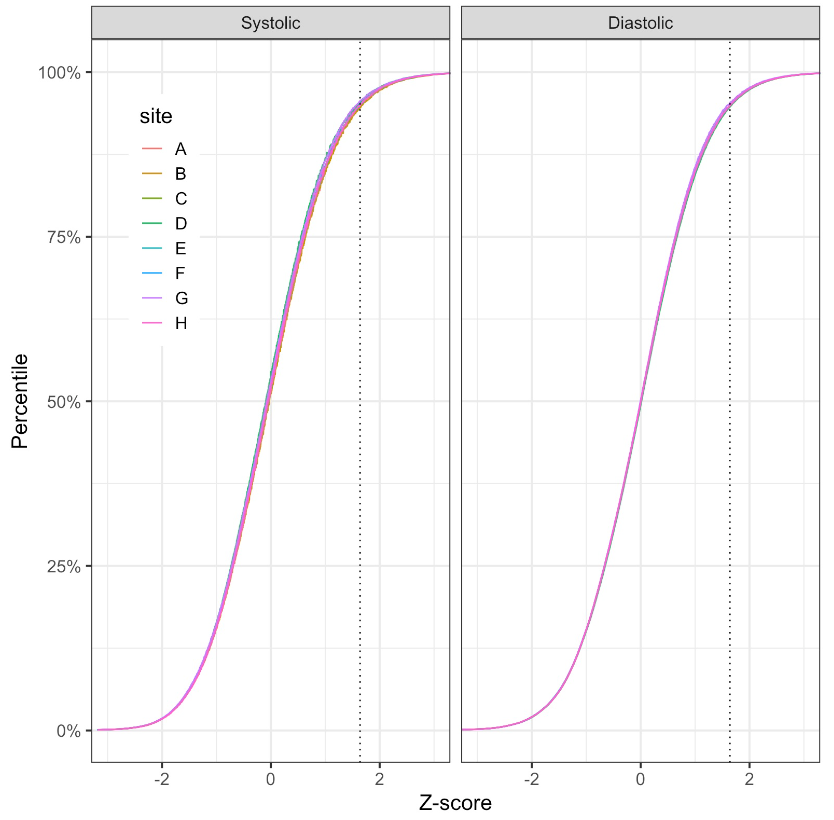
 **c. Health Systems**

**Supplementary Figure S2a-d: Systolic and diastolic BP at the 90^th^ percentile by age and different height percentiles for boys and girls.** BP increase with age was similar for different height percentiles.

a.

b.


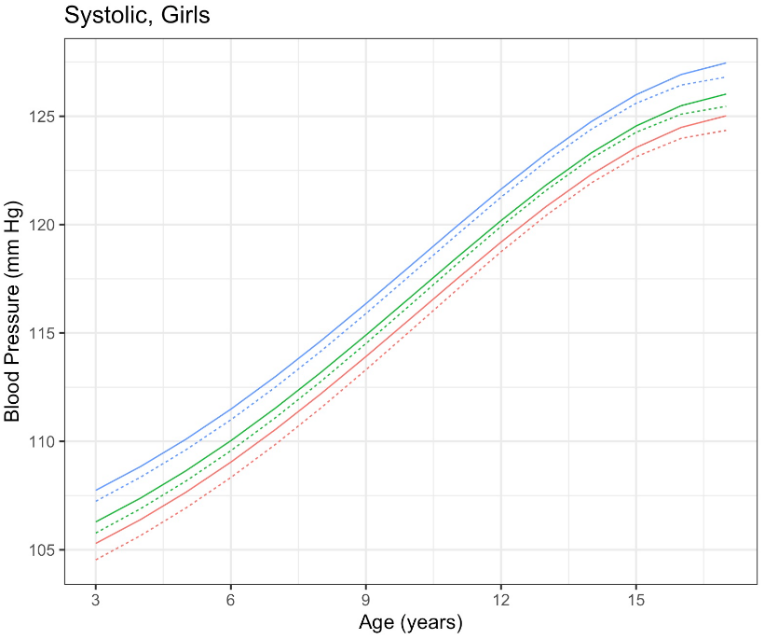

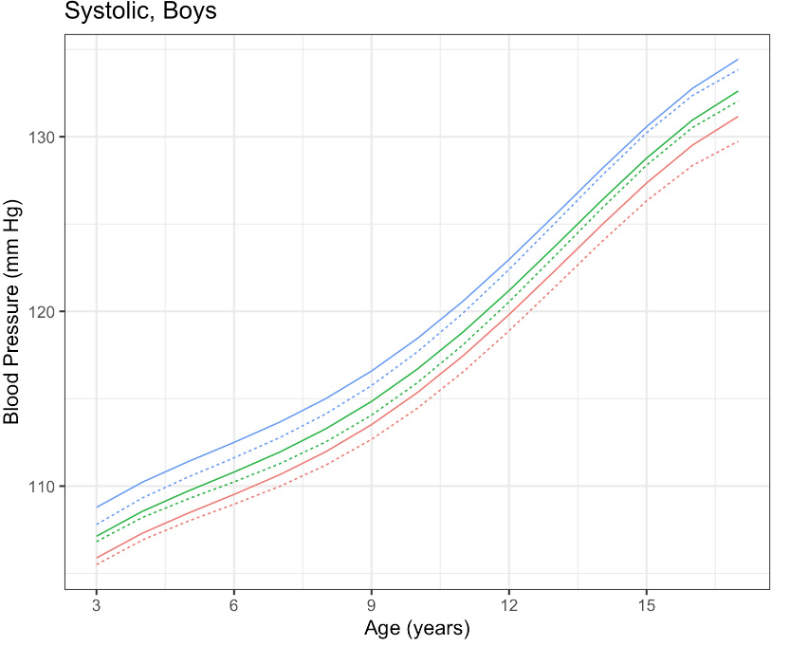


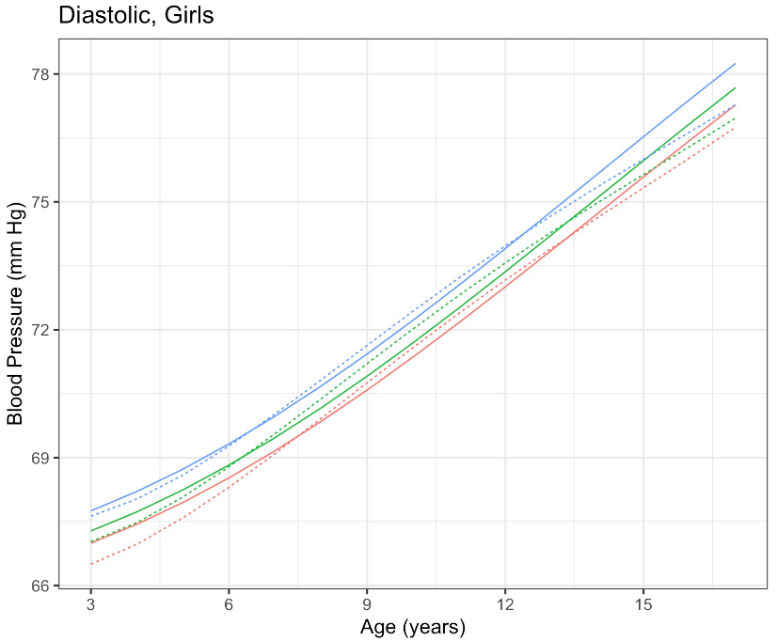

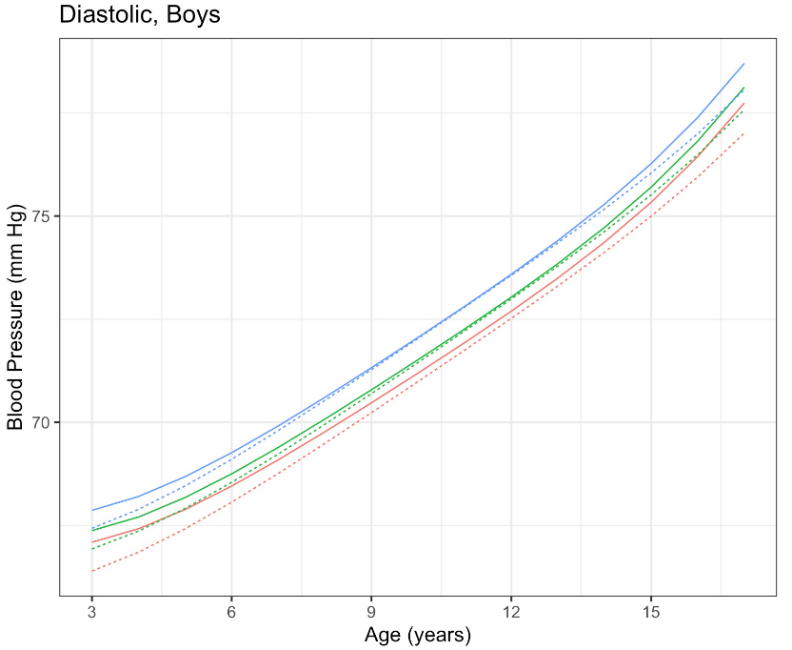


d..

c..


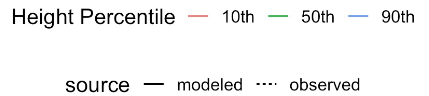


**Supplementary Table S1. Sample size derivation by site**

|  | **Total** | **CCHMC** | **CHOP** | **Colorado** | **Lurie** | **Nationwide** | **Nemours** | **Seattle** | **Stanford** |
| --- | --- | --- | --- | --- | --- | --- | --- | --- | --- |
| All persons in PEDSnet database | 8,178,890 | 949,978 | 1,432,705 | 1,236,104 | 814,302 | 1,004,938 | 1,553,643 | 714,964 | 472,256 |
| Physician visits made to outpatient settings between Jan 1, 2009 and Aug 31, 2021 | 6,732,440 | 841,406 | 1,260,956 | 875,109 | 535,678 | 808,615 | 1,451,346 | 534,179 | 425,151 |
| At least 1 year of follow-up | 3,423,636 | 494,289 | 771,885 | 351,084 | 204,635 | 496,715 | 683,813 | 221,068/ | 200,14 |
| *Either ≤3 years follow-up or general pediatrics visits every 18 months | 1,844,018 | 134,568 | 570,743 | 150,154 | 93,723 | 248,145 | 409,803 | 84,821 | 152,061 |
| Age ≥3 and <18 years of age on qualified visit date | 1,489,523 | 86,353 | 492,873 | 113,827 | 71,690 | 190,776 | 345,524 | 65,957 | 122,523 |
| Systolic and Diastolic BP on the same day at the same encounter | 1,212,978 | 48,811 | 446,581 | 82,274 | 49,100 | 151,256 | 287,199 | 40,834 | 106,923 |
| Exclude BPs within 30 days of outpatient surgery with general anesthesia | 1,201,609 | 48,792 | 442,879 | 77,756 | 49,098 | 150,952 | 286,488 | 38,906 | 106,738 |
| BPs with height within 90 days and height z-scores <-3.09 or > +3.09 | 1,167,090 | 45,775 | 438,067 | 75,644 | 47,259 | 147,003 | 275,552 | 31,829 | 105,961 |
| BMI at any time | 1,167,064 | 45,768 | 438,063 | 75,640 | 47,258 | 146,999 | 275,552 | 31,826 | 105,958 |
| No BMI ≥ 85th percentile at any time | 578,799 | 26,122 | 223,530 | 43,568 | 24,157 | 63,611 | 117,670 | 18,305 | 61,836 |
| **No chronic condition at any time | 347,360 | 18,008 | 138,259 | 26,198 | 14,411 | 38,171 | 64,778 | 11,269 | 36,266 |
| No diagnosis of hypertension or exposure to anti-hypertensive medications | 339,799 | 17,426 | 135,338 | 25,362 | 13,868 | 37,340 | 63,601 | 10,887 | 35,977 |
| No exposure to systemic corticosteroids or stimulants | 292,412 | 16,366 | 105,860 | 23,831 | 12,689 | 32,950 | 56,555 | 10,478 | 33,683 |

*****The general pediatric visit defined as at least two outpatient general practice or family medicine encounters and at least one such encounter every 18 months if followed >3 years (approximating recommendations for frequency of well-child visits).

**Chronic condition was defined based on the Pediatric Medical Complexity Algorithm (PMCA). (9,10)

BP, blood pressure; BMI, body mass index

**Supplementary Table S2: BP (mmHg) values for Z-scores (-5/5+) by age, sex, and height 50^th^ percentile**

| **Age, years** | **Boys** | | | | **Girls** | | | |
| --- | --- | --- | --- | --- | --- | --- | --- | --- |
|  | **Z-score -5** | | **Z-score 5+** | | **Z-score -5** | | **Z-score 5+** | |
|  | **Systolic BP** | **Diastolic BP** | **Systolic BP** | **Diastolic BP** | **Systolic BP** | **Diastolic BP** | **Systolic BP** | **Diastolic BP** |
| 3 | 49.1 | 21.5 | 141.5 | 94.5 | 49.1 | 22.1 | 140.2 | 94.1 |
| 4 | 51.0 | 22.7 | 142.6 | 94.3 | 50.6 | 23.3 | 141.0 | 94.0 |
| 5 | 52.5 | 23.8 | 143.5 | 94.4 | 52.1 | 24.4 | 142.1 | 94.1 |
| 6 | 53.7 | 24.9 | 144.5 | 94.7 | 53.6 | 25.4 | 143.5 | 94.5 |
| 7 | 54.8 | 25.8 | 145.7 | 95.1 | 55.0 | 26.3 | 145.0 | 95.0 |
| 8 | 55.8 | 26.6 | 147.2 | 95.8 | 56.3 | 27.0 | 146.8 | 95.7 |
| 9 | 57.0 | 27.3 | 149.1 | 96.6 | 57.6 | 27.6 | 148.8 | 96.5 |
| 10 | 58.2 | 27.7 | 151.4 | 97.5 | 58.8 | 28.1 | 150.9 | 97.5 |
| 11 | 59.5 | 28.0 | 154.0 | 98.5 | 59.8 | 28.4 | 153.2 | 98.6 |
| 12 | 60.9 | 28.0 | 157.0 | 99.7 | 60.6 | 28.6 | 155.4 | 99.8 |
| 13 | 62.3 | 28.0 | 160.2 | 101.0 | 61.2 | 28.6 | 157.7 | 101.2 |
| 14 | 63.5 | 27.8 | 163.5 | 102.5 | 61.5 | 28.4 | 159.9 | 102.7 |
| 15 | 64.5 | 27.6 | 166.9 | 104.2 | 61.4 | 28.2 | 161.9 | 104.2 |
| 16 | 65.0 | 27.4 | 170.0 | 106.1 | 60.9 | 27.8 | 163.7 | 105.9 |
| 17 | 64.9 | 27.2 | 172.6 | 108.2 | 59.9 | 27.3 | 165.2 | 107.6 |

**Supplementary Table S3. Demographic characteristics by health system**

|  | **Total** | **CCHMC** | **CHOP** | **Colorado** | **Lurie** | **Nationwide** | **Nemours** | **Seattle** | **Stanford** |
| --- | --- | --- | --- | --- | --- | --- | --- | --- | --- |
| ***Age (years)** | 9.9  (5.8 -14.3) | 12.6  (7.1 - 15.9) | 9.3  (5.5 - 13.8) | 10.4  (5.9 - 15.0) | 11.1  (6.4 - 14.8) | 9.0  (5.2 - 14.1) | 10.2  (6.1 - 14.3) | 13.9  (8.7 - 16.3) | 10.0  (6.1 - 13.8) |
| **Sex** |  |  |  |  |  |  |  |  |  |
| Girls | 154,814 (52.9%) | 8,069 (49.3%) | 57,416 (54.2%) | 12,224 (51.3%) | 6,450 (50.8%) | 17,619  (53.5%) | 29,732 (52.6%) | 5,385  (51.4%) | 17,919 (53.2%) |
| Boys | 137,598 (47.1%) | 8,297 (50.7%) | 48,444 (45.8%) | 11,607 (48.7%) | 6,239 (49.2%) | 15,331  (46.5%) | 26,823 (47.4%) | 5,093  (48.6%) | 15,764 (46.8%) |
| **Race/Ethnicity** |  |  |  |  |  |  |  |  |  |
| Asian/PI | 19,725 (6.7%) | 379  2.3%) | 5,869  (5.5%) | 737  (3.1%) | 846  (6.7%) | 2,322  (7.0%) | 2,832  (5.0%) | 936  (8.9%) | 5,804 (17.2%) |
| Black/AA | 55,252 (18.9%) | 1,894 (11.6%) | 24,254 (22.9%) | 2,156 (9.0%) | 1,595 (12.6%) | 14,176  (43.0%) | 9,899 (17.5%) | 592  (5.6%) | 686  (2.0%) |
| Hispanic | 30,484 (10.4%) | 622  (3.8%) | 6,153  (5.8%) | 6,562 (27.5%) | 2,860 (22.5%) | 2,802  (8.5%) | 7,464 (13.2%) | 1,279  (12.2%) | 2,742 (8.1%) |
| Multiple | 6,844 (2.3%) | 364  (2.2%) | 1,500  (1.4%) | 1,154 (4.8%) | 175  (1.4%) | 1,689  (5.1%) | 926  (1.6%) | 29  (0.3%) | 1,007 (3.0%) |
| Other/Unknown | 38,814 (13.3%) | 674  (4.1%) | 12,722 (12.0%) | 2,016 (8.5%) | 594  (4.7%) | 672  (2.0%) | 5,842 (10.3%) | 1,656  (15.8%) | 14,638 (43.5%) |
| White | 141,293 (48.3%) | 12,433 (76.0%) | 55,362 (52.3%) | 11,206 (47.0%) | 6,619 (52.2%) | 11,289  (34.3%) | 29,592 (52.3%) | 5,986  (57.1%) | 8,806 (26.1%) |

*Data presented as median (IQR). Age assessed across all measurement events.

**Supplementary Table S4. Number of children contributed to analysis by age and health system.**

| **Age (years)** | **Total** | **CCHMC** | **CHOP** | **Colorado** | **Lurie** | **Nationwide** | **Nemours** | **Seattle** | **Stanford** |
| --- | --- | --- | --- | --- | --- | --- | --- | --- | --- |
| 3 | 74,296 (25.4%) | 2,076 (12.7%) | 36,068 (34.1%) | 4,391 (18.4%) | 2,281 (18.0%) | 10,036 (30.5%) | 11,311 (20.0%) | 1,076 (10.3%) | 7,057 (21.0%) |
| 4 | 74,105 (25.3%) | 1,649 (10.1%) | 34,987 (33.1%) | 4,145 (17.4%) | 2,032 (16.0%) | 9,859 (29.9%) | 12,018 (21.3%) | 920 (8.8%) | 8,495 (25.2%) |
| 5 | 67,302 (23.0%) | 1,410 (8.6%) | 32,299 (30.5%) | 3,508 (14.7%) | 1,906 (15.0%) | 8,045 (24.4%) | 11,593 (20.5%) | 895 (8.5%) | 7,646 (22.7%) |
| 6 | 57,653 (19.7%) | 1,292 (7.9%) | 27,599 (26.1%) | 2,944 (12.4%) | 1,669 (13.2%) | 6,151 (18.7%) | 9,969 (17.6%) | 881 (8.4%) | 7,148 (21.2%) |
| 7 | 52,279 (17.9%) | 1,155 (7.1%) | 25,174 (23.8%) | 2,722 (11.4%) | 1,596 (12.6%) | 5,106 (15.5%) | 9,329 (16.5%) | 817 (7.8%) | 6,380 (18.9%) |
| 8 | 48,878 (16.7%) | 1,059 (6.5%) | 23,523 (22.2%) | 2,478 (10.4%) | 1,494 (11.8%) | 4,548 (13.8%) | 8,803 (15.6%) | 757 (7.2%) | 6,216 (18.5%) |
| 9 | 46,404 (15.9%) | 1,020 (6.2%) | 22,315 (21.1%) | 2,249 (9.4%) | 1,502 (11.8%) | 4,186 (12.7%) | 8,441 (14.9%) | 771 (7.4%) | 5,920 (17.6%) |
| 10 | 44,806 (15.3%) | 1,014 (6.2%) | 21,443 (20.3%) | 2,122 (8.9%) | 1,440 (11.3%) | 3,995 (12.1%) | 8,151 (14.4%) | 782 (7.5%) | 5,859 (17.4%) |
| 11 | 49,671 (17.0%) | 1,126 (6.9%) | 24,353 (23.0%) | 2,224 (9.3%) | 1,670 (13.2%) | 4,031 (12.2%) | 8,883 (15.7%) | 846 (8.1%) | 6,538 (19.4%) |
| 12 | 48,193 (16.5%) | 1,247 (7.6%) | 22,900 (21.6%) | 2,265 (9.5%) | 1,665 (13.1%) | 4,377 (13.3%) | 8,834 (15.6%) | 863 (8.2%) | 6,042 (17.9%) |
| 13 | 48,011 (16.4%) | 1,461 (8.9%) | 22,505 (21.3%) | 2,369 (9.9%) | 1,890 (14.9%) | 4,302 (13.1%) | 8,751 (15.5%) | 1,122 (10.7%) | 5,611 (16.7%) |
| 14 | 50,504 (17.3%) | 1,803 (11.0%) | 22,996 (21.7%) | 2,647 (11.1%) | 2,156 (17.0%) | 4,577 (13.9%) | 9,067 (16.0%) | 1,417 (13.5%) | 5,841 (17.3%) |
| 15 | 51,959 (17.8%) | 2,129 (13.0%) | 23,245 (22.0%) | 3,041 (12.8%) | 2,105 (16.6%) | 4,875 (14.8%) | 9,185 (16.2%) | 1,806 (17.2%) | 5,573 (16.5%) |
| 16 | 52,384 (17.9%) | 2,551 (15.6%) | 24,039 (22.7%) | 3,093 (13.0%) | 2,076 (16.4%) | 4,907 (14.9%) | 8,686 (15.4%) | 1,893 (18.1%) | 5,139 (15.3%) |
| 17 | 46,021 (15.7%) | 2,370 (14.5%) | 20,925 (19.8%) | 2,855 (12.0%) | 1,812 (14.3%) | 4,617 (14.0%) | 7,230 (12.8%) | 1,805 (17.2%) | 4,407 (13.1%) |

**Supplementary Table S5. Regressions between observation and model prediction for the test and training sets.** Intercept and slope are consistent within the limits of the standard error of the estimate.

|  | **Sex category** | **Set** | **Term** | **Estimate** | **Standard error** |
| --- | --- | --- | --- | --- | --- |
| **Systolic BP** | **Boys** | test | (Intercept) | 0.802 | 0.333 |
|  |  | training | (Intercept) | 0.881 | 0.218 |
|  |  | test | slope | 0.989 | 0.003 |
|  |  | training | slope | 0.988 | 0.002 |
|  |  | test | R-squared | 0.401 |  |
|  |  | training | R-squared | 0.401 |  |
|  | **Girls** | test | (Intercept) | 0.855 | 0.356 |
|  |  | training | (Intercept) | 1.818 | 0.233 |
|  |  | test | slope | 0.990 | 0.003 |
|  |  | training | slope | 0.980 | 0.002 |
|  |  | test | R-squared | 0.319 |  |
|  |  | training | R-squared | 0.316 |  |
| **Diastolic BP** | **Boys** | test | (Intercept) | 4.631 | 0.384 |
|  |  | training | (Intercept) | 4.131 | 0.249 |
|  |  | test | slope | 0.926 | 0.006 |
|  |  | training | slope | 0.934 | 0.004 |
|  |  | test | R-squared | 0.133 |  |
|  |  | training | R-squared | 0.137 |  |
|  | **Girls** | test | (Intercept) | 4.404 | 0.337 |
|  |  | training | (Intercept) | 5.491 | 0.220 |
|  |  | test | slope | 0.931 | 0.005 |
|  |  | training | slope | 0.913 | 0.004 |
|  |  | test | R-squared | 0.143 |  |
|  |  | training | R-squared | 0.140 |  |

**Supplementary Table S6. Comparisons of BP classifications between new model and 2017 AAP CPG**

| **3–17-year-old** | |  |  |  |  |  |  |
| --- | --- | --- | --- | --- | --- | --- | --- |
| **BP Type** | **2017 AAP CPG classification** | **<10^th^ %tile** | **10-90^th^ %tile** | **90-95^th^ %tile** | **95-95^th^ %tile +12 mmHg** | **>95^th^ %tile +12 mmHg** | **2017 AAP CPG**  **n (%)** |
| **Systolic** | **<10%tile** | 21,824 | 919 | 0 | 0 | 0 | 22,743 (2.1`) |
|  | **10-90^th^ %tile** | 26,330 | 920,352 | 4,968 | 0 | 0 | 951,650 (87.7) |
|  | **90-95^th^ %tile** | 0 | 16,591 | 33,885 | 4,132 | 0 | 54,608 (5.0) |
|  | **95-95^th^ %tile**  **+ 12 mmHg** | 0 | 278 | 6,632 | 43,006 | 439 | 50,355 (4.6) |
|  | **>95^th^ %tile**  **+12 mmHg** | 0 | 0 | 0 | 861 | 4,866 | 5,727 (0.5) |
|  | **PEDSnet, n (%)** | 48,154 (4.4) | 938,140 (86.5) | 45,485 (4.2) | 47,499 (4.4) | 5,305 (0.5) |  |
| **Diastolic** | **<10%tile** | 3,341 | 0 | 0 | 0 | 0 | 3,341 (0.3) |
|  | **10-90^th^ %tile** | 44,876 | 918,140 | 37,098 | 10,557 | 0 | 1,010,671 (93.1) |
|  | **90-95^th^ %tile** | 0 | 13,879 | 8,931 | 23,152 | 0 | 45,962 (4.2) |
|  | **95-95^th^ %tile**  **+ 12 mmHg** | 0 | 1,065 | 2,853 | 18,522 | 1,170 | 23,610 (2.2) |
|  | **>95^th^ %tile**  **+12 mmHg** | 0 | 0 | 0 | 281 | 1,218 | 1,499 (0.1) |
|  | **PEDSnet, n (%)** | 48,217 (4.4) | 933,084 (85.0) | 48,882 (4.5) | 51,028 (4.7) | 2,388 (0.2) |  |

| **3–12-year-old** | |  |  |  |  |  |  |
| --- | --- | --- | --- | --- | --- | --- | --- |
| **BP Type** | **2017 AAP CPG classification** | **<10^th^ %tile** | **10-90^th^ %tile** | **90-95^th^ %tile** | **95-95^th^ %tile +12 mmHg** | **>95^th^ %tile +12 mmHg** | **2017 AAP CPG**  **n (%)** |
| **Systolic** | **<10%tile** | 8,600 | 0 | 0 | 0 | 0 | 8,600 (1.2) |
|  | **10-90^th^ %tile** | 21,915 | 621,618 | 2,717 | 0 | 0 | 646,250 (88.4) |
|  | **90-95^th^ %tile** | 0 | 13,036 | 22,615 | 2,668 | 0 | 38,319 (5,2) |
|  | **95-95^th^ %tile**  **+ 12 mmHg** | 0 | 224 | 4,814 | 28,833 | 648 | 34,519 (4.7) |
|  | **>95^th^ %tile**  **+ 12 mmHg** | 0 | 0 | 0 | 361 | 3,345 | 3,706 (0.5) |
|  | **PEDSnet n (%)** | 30,515 (4.2) | 634,878 (86.8) | 30,146 (4.1) | 31,862 (4.4) | 3,993 (0.5) |  |
| **Diastolic** | **<10%tile** | 1,101 | 0 | 0 | 0 | 0 | 1,101 (0.2) |
|  | **10-90th %tile** | 30,511 | 613,952 | 21,710 | 5,677 | 0 | 671,850 (91.9) |
|  | **90-95th %tile** | 0 | 13,879 | 8,446 | 15,068 | 0 | 37,393 (5.1) |
|  | **95-95th %tile**  **+ 12 mmHg** | 0 | 1,065 | 2,853 | 14,081 | 1,894 | 19,893 (2.7) |
|  | **>95th %tile**  **+12 mmHg** | 0 | 0 | 0 | 60 | 1,097 | 1,157 (0.2) |
|  | **PEDSnet n (%)** | 31,612 (4.3) | 628,896 (86.0) | 33,009 (4.5) | 34,886 (4.8) | 2,991 (0.4) |  |
|  |  |  |  |  |  |  |  |

**Supplementary Table S7. BP Levels for Boys by Age and Height Percentile**

|  | **BP percentile** | **Systolic BP** | | | | | | | **Diastolic BP** | | | | | | |
| --- | --- | --- | --- | --- | --- | --- | --- | --- | --- | --- | --- | --- | --- | --- | --- |
|  |  | **Height Percentile** | | | | | | | **Height Percentile** | | | | | | |
|  |  | **5^th^** | **10^th^** | **25^th^** | **50^th^** | **75^th^** | **90^th^** | **95^th^** | **5th** | **10th** | **25th** | **50th** | **75th** | **90th** | **95th** |
| 3 | height (in) | 36.4 | 37.0 | 37.9 | 39.0 | 40.1 | 41.1 | 41.7 | 36.4 | 37.0 | 37.9 | 39.0 | 40.1 | 41.1 | 41.7 |
|  | height (cm) | 92.5 | 93.9 | 96.3 | 99.0 | 101.8 | 104.3 | 105.8 | 92.5 | 93.9 | 96.3 | 99.0 | 101.8 | 104.3 | 105.8 |
|  | 50th | 93.5 | 93.8 | 94.5 | 95.3 | 96.1 | 96.9 | 97.3 | 57.5 | 57.6 | 57.8 | 58.0 | 58.3 | 58.5 | 58. |
|  | 75th | 99.9 | 100.2 | 100.7 | 101.5 | 102.4 | 103.1 | 103.6 | 62.5 | 62.6 | 62.7 | 62.9 | 63.2 | 63.4 | 63.6 |
|  | 90th | 105.7 | 105.9 | 106.4 | 107.1 | 108.0 | 108.8 | 109.3 | 67.1 | 67.1 | 67.2 | 67.4 | 67.6 | 67.9 | 68.1 |
|  | 95th | 109.2 | 109.3 | 109.8 | 110.5 | 111.3 | 112.2 | 112.7 | 69.8 | 69.8 | 69.9 | 70.0 | 70.3 | 70.5 | 70.7 |
|  |  |  |  |  |  |  |  |  |  |  |  |  |  |  |  |
| 4 | height (in) | 38.8 | 39.4 | 40.5 | 41.7 | 42.9 | 43.9 | 44.5 | 38.8 | 39.4 | 40.5 | 41.7 | 42.9 | 43.9 | 44.5 |
|  | height (cm) | 98.5 | 100.2 | 102.9 | 105.9 | 108.9 | 111.6 | 113.2 | 98.5 | 100.2 | 102.9 | 105.9 | 108.9 | 111.6 | 113.2 |
|  | 50th | 95.0 | 95.3 | 96.0 | 96.8 | 97.7 | 98.4 | 98.9 | 58.0 | 58.1 | 58.3 | 58.5 | 58.8 | 59.0 | 59.2 |
|  | 75th | 101.4 | 101.6 | 102.2 | 103.0 | 103.8 | 104.6 | 105.1 | 62.9 | 63.0 | 63.1 | 63.4 | 63.6 | 63.8 | 64.0 |
|  | 90th | 107.1 | 107.3 | 107.8 | 108.6 | 109.4 | 110.2 | 110.8 | 67.4 | 67.4 | 67.5 | 67.7 | 67.9 | 68.2 | 68.4 |
|  | 95th | 110.5 | 110.7 | 111.2 | 111.9 | 112.7 | 113.6 | 114.1 | 70.1 | 70.1 | 70.1 | 70.3 | 70.5 | 70.8 | 71.0 |
|  |  |  |  |  |  |  |  |  |  |  |  |  |  |  |  |
| 5 | height (in) | 41.1 | 41.8 | 43.0 | 44.3 | 45.5 | 46.7 | 47.4 | 41.1 | 41.8 | 43.0 | 44.3 | 45.5 | 46.7 | 47.4 |
|  | height (cm) | 104.4 | 106.2 | 109.1 | 112.4 | 115.7 | 118.6 | 120.3 | 104.4 | 106.2 | 109.1 | 112.4 | 115.7 | 118.6 | 120.3 |
|  | 50th | 96.2 | 96.5 | 97.2 | 98.0 | 98.9 | 99.6 | 100.1 | 58.6 | 58.7 | 58.9 | 59.1 | 59.4 | 59.6 | 59.8 |
|  | 75th | 102.6 | 102.8 | 103.4 | 104.2 | 105.0 | 105.8 | 106.3 | 63.5 | 63.5 | 63.7 | 63.9 | 64.1 | 64.4 | 64.6 |
|  | 90th | 108.3 | 108.5 | 109.0 | 109.7 | 110.6 | 111.4 | 112.0 | 67.9 | 67.9 | 68.0 | 68.2 | 68.4 | 68.7 | 68.9 |
|  | 95th | 111.7 | 111.8 | 112.3 | 113.0 | 113.9 | 114.7 | 115.3 | 70.5 | 70.5 | 70.6 | 70.7 | 71.0 | 71.3 | 71.5 |
|  |  |  |  |  |  |  |  |  |  |  |  |  |  |  |  |
| 6 | height (in) | 43.4 | 44.2 | 45.4 | 46.8 | 48.2 | 49.4 | 50.2 | 43.4 | 44.2 | 45.4 | 46.8 | 48.2 | 49.4 | 50.2 |
|  | height (cm) | 110.3 | 112.2 | 115.3 | 118.9 | 122.4 | 125.6 | 127.5 | 110.3 | 112.2 | 115.3 | 118.9 | 122.4 | 125.6 | 127.5 |
|  | 50th | 97.3 | 97.7 | 98.3 | 99.2 | 100.0 | 100.7 | 101.2 | 59.3 | 59.4 | 59.6 | 59.8 | 60.1 | 60.3 | 60.5 |
|  | 75th | 103.6 | 103.9 | 104.5 | 105.3 | 106.1 | 106.9 | 107.4 | 64.1 | 64.2 | 64.3 | 64.5 | 64.8 | 65.0 | 65.2 |
|  | 90th | 109.3 | 109.5 | 110.0 | 110.8 | 111.7 | 112.5 | 113.1 | 68.4 | 68.5 | 68.6 | 68.8 | 69.0 | 69.3 | 69.5 |
|  | 95th | 112.7 | 112.9 | 113.4 | 114.1 | 115.0 | 115.8 | 116.4 | 71.0 | 71.0 | 71.1 | 71.3 | 71.5 | 71.8 | 72.0 |
|  |  |  |  |  |  |  |  |  |  |  |  |  |  |  |  |
| 7 | height (in) | 45.7 | 46.5 | 47.8 | 49.3 | 50.8 | 52.1 | 52.9 | 45.7 | 46.5 | 47.8 | 49.3 | 50.8 | 52.1 | 52.9 |
|  | height (cm) | 116.1 | 118.0 | 121.4 | 125.1 | 128.9 | 132.4 | 134.5 | 116.1 | 118.0 | 121.4 | 125.1 | 128.9 | 132.4 | 134.5 |
|  | 50th | 98.5 | 98.8 | 99.5 | 100.3 | 101.1 | 101.9 | 102.3 | 60.0 | 60.1 | 60.3 | 60.5 | 60.8 | 61.0 | 61.2 |
|  | 75th | 104.8 | 105.0 | 105.6 | 106.4 | 107.3 | 108.1 | 108.6 | 64.8 | 64.8 | 65.0 | 65.2 | 65.4 | 65.7 | 65.9 |
|  | 90th | 110.5 | 110.7 | 111.2 | 112.0 | 112.8 | 113.7 | 114.2 | 69.1 | 69.1 | 69.2 | 69.4 | 69.6 | 69.9 | 70.1 |
|  | 95th | 113.9 | 114.0 | 114.5 | 115.3 | 116.1 | 117.0 | 117.6 | 71.6 | 71.7 | 71.7 | 71.9 | 72.2 | 72.4 | 72.7 |
|  |  |  |  |  |  |  |  |  |  |  |  |  |  |  |  |
| 8 | height (in) | 47.8 | 48.6 | 50.0 | 51.6 | 53.2 | 54.6 | 55.5 | 47.8 | 48.6 | 50.0 | 51.6 | 53.2 | 54.6 | 55.5 |
|  | height (cm) | 121.4 | 123.5 | 127.0 | 131.0 | 135.1 | 138.8 | 141.0 | 121.4 | 123.5 | 127.0 | 131.0 | 135.1 | 138.8 | 141.0 |
|  | 50th | 99.7 | 100.1 | 100.7 | 101.6 | 102.4 | 103.1 | 103.6 | 60.7 | 60.8 | 61.0 | 61.2 | 61.5 | 61.7 | 61.9 |
|  | 75th | 106.1 | 106.3 | 106.9 | 107.7 | 108.6 | 109.4 | 109.9 | 65.5 | 65.5 | 65.7 | 65.9 | 66.1 | 66.4 | 66.6 |
|  | 90th | 111.8 | 112.0 | 112.5 | 113.3 | 114.2 | 115.0 | 115.6 | 69.7 | 69.8 | 69.9 | 70.1 | 70.3 | 70.6 | 70.8 |
|  | 95th | 115.2 | 115.3 | 115.8 | 116.6 | 117.5 | 118.4 | 119.0 | 72.3 | 72.3 | 72.4 | 72.6 | 72.8 | 73.1 | 73.4 |
|  |  |  |  |  |  |  |  |  |  |  |  |  |  |  |  |
| 9 | height (in) | 49.6 | 50.5 | 52.0 | 53.7 | 55.4 | 57.0 | 57.9 | 49.6 | 50.5 | 52.0 | 53.7 | 55.4 | 57.0 | 57.9 |
|  | height (cm) | 126.0 | 128.3 | 132.1 | 136.3 | 140.7 | 144.7 | 147.1 | 126.0 | 128.3 | 132.1 | 136.3 | 140.7 | 144.7 | 147.1 |
|  | 50th | 101.2 | 101.5 | 102.2 | 103.0 | 103.9 | 104.6 | 105.1 | 61.4 | 61.5 | 61.7 | 61.9 | 62.2 | 62.4 | 62.6 |
|  | 75th | 107.6 | 107.8 | 108.5 | 109.3 | 110.1 | 110.9 | 111.4 | 66.1 | 66.2 | 66.4 | 66.6 | 66.8 | 67.1 | 67.3 |
|  | 90th | 113.3 | 113.5 | 114.1 | 114.8 | 115.7 | 116.6 | 117.2 | 70.4 | 70.5 | 70.6 | 70.8 | 71.0 | 71.3 | 71.5 |
|  | 95th | 116.7 | 116.9 | 117.4 | 118.2 | 119.1 | 120.0 | 120.6 | 73.0 | 73.0 | 73.1 | 73.3 | 73.6 | 73.9 | 74.1 |
|  |  |  |  |  |  |  |  |  |  |  |  |  |  |  |  |
| 10 | height (in) | 51.3 | 52.2 | 53.8 | 55.6 | 57.4 | 59.1 | 60.1 | 51.3 | 52.2 | 53.8 | 55.6 | 57.4 | 59.1 | 60.1 |
|  | height (cm) | 130.2 | 132.6 | 136.7 | 141.3 | 145.9 | 150.1 | 152.7 | 130.2 | 132.6 | 136.7 | 141.3 | 145.9 | 150.1 | 152.7 |
|  | 50th | 102.9 | 103.3 | 103.9 | 104.8 | 105.6 | 106.3 | 106.8 | 62.0 | 62.1 | 62.3 | 62.6 | 62.8 | 63.1 | 63.2 |
|  | 75th | 109.4 | 109.6 | 110.2 | 111.0 | 111.9 | 112.7 | 113.2 | 66.8 | 66.9 | 67.1 | 67.3 | 67.5 | 67.8 | 68.0 |
|  | 90th | 115.1 | 115.4 | 115.9 | 116.7 | 117.6 | 118.5 | 119.0 | 71.2 | 71.2 | 71.3 | 71.5 | 71.8 | 72.1 | 72.3 |
|  | 95th | 118.6 | 118.8 | 119.3 | 120.1 | 121.0 | 121.9 | 122.5 | 73.8 | 73.8 | 73.9 | 74.1 | 74.3 | 74.6 | 74.8 |
|  |  |  |  |  |  |  |  |  |  |  |  |  |  |  |  |
| 11 | height (in) | 53.0 | 54.0 | 55.7 | 57.6 | 59.6 | 61.3 | 62.4 | 53.0 | 54.0 | 55.7 | 57.6 | 59.6 | 61.3 | 62.4 |
|  | height (cm) | 134.7 | 137.2 | 141.5 | 146.4 | 151.3 | 155.8 | 158.6 | 134.7 | 137.2 | 141.5 | 146.4 | 151.3 | 155.8 | 158.6 |
|  | 50th | 104.9 | 105.2 | 105.9 | 106.7 | 107.6 | 108.3 | 108.8 | 62.7 | 62.8 | 63.0 | 63.2 | 63.5 | 63.7 | 63.9 |
|  | 75th | 111.4 | 111.7 | 112.3 | 113.1 | 114.0 | 114.8 | 115.3 | 67.5 | 67.6 | 67.8 | 68.0 | 68.2 | 68.5 | 68.7 |
|  | 90th | 117.2 | 117.5 | 118.0 | 118.8 | 119.7 | 120.6 | 121.2 | 71.9 | 71.9 | 72.1 | 72.3 | 72.5 | 72.8 | 73.0 |
|  | 95th | 120.7 | 120.9 | 121.5 | 122.3 | 123.2 | 124.1 | 124.7 | 74.5 | 74.5 | 74.6 | 74.8 | 75.1 | 75.4 | 75.6 |
|  |  |  |  |  |  |  |  |  |  |  |  |  |  |  |  |
| 12 | height (in) | 55.2 | 56.3 | 58.1 | 60.1 | 62.2 | 64.0 | 65.2 | 55.2 | 56.3 | 58.1 | 60.1 | 62.2 | 64.0 | 65.2 |
|  | height (cm) | 140.3 | 143.0 | 147.5 | 152.7 | 157.9 | 162.6 | 165.5 | 140.3 | 143.0 | 147.5 | 152.7 | 157.9 | 162.6 | 165.5 |
|  | 50th | 107.1 | 107.4 | 108.1 | 108.9 | 109.7 | 110.5 | 110.9 | 63.3 | 63.4 | 63.6 | 63.9 | 64.1 | 64.3 | 64.5 |
|  | 75th | 113.7 | 113.9 | 114.5 | 115.4 | 116.2 | 117.1 | 117.6 | 68.2 | 68.3 | 68.5 | 68.7 | 68.9 | 69.2 | 69.4 |
|  | 90th | 119.6 | 119.8 | 120.4 | 121.2 | 122.1 | 123.0 | 123.5 | 72.7 | 72.7 | 72.8 | 73.0 | 73.3 | 73.6 | 73.8 |
|  | 95th | 123.1 | 123.3 | 123.9 | 124.7 | 125.6 | 126.5 | 127.1 | 75.3 | 75.3 | 75.4 | 75.6 | 75.9 | 76.2 | 76.4 |
|  |  |  |  |  |  |  |  |  |  |  |  |  |  |  |  |
| 13 | height (in) | 57.9 | 59.1 | 61.0 | 63.1 | 65.2 | 67.1 | 68.3 | 57.9 | 59.1 | 61.0 | 63.1 | 65.2 | 67.1 | 68.3 |
|  | height (cm) | 147.0 | 150.0 | 154.9 | 160.3 | 165.7 | 170.5 | 173.4 | 147.0 | 150.0 | 154.9 | 160.3 | 165.7 | 170.5 | 173.4 |
|  | 50th | 109.4 | 109.7 | 110.3 | 111.2 | 112.0 | 112.8 | 113.2 | 63.9 | 64.0 | 64.2 | 64.5 | 64.7 | 65.0 | 65.1 |
|  | 75th | 116.1 | 116.3 | 117.0 | 117.8 | 118.7 | 119.5 | 120.0 | 69.0 | 69.0 | 69.2 | 69.4 | 69.7 | 69.9 | 70.1 |
|  | 90th | 122.1 | 122.3 | 122.9 | 123.7 | 124.6 | 125.5 | 126.1 | 73.5 | 73.5 | 73.6 | 73.8 | 74.1 | 74.4 | 74.6 |
|  | 95th | 125.7 | 125.9 | 126.5 | 127.3 | 128.2 | 129.1 | 129.7 | 76.2 | 76.2 | 76.3 | 76.5 | 76.8 | 77.1 | 77.3 |
|  |  |  |  |  |  |  |  |  |  |  |  |  |  |  |  |
| 14 | height (in) | 60.6 | 61.8 | 63.8 | 65.9 | 68.0 | 69.8 | 70.9 | 60.6 | 61.8 | 63.8 | 65.9 | 68.0 | 69.8 | 70.9 |
|  | height (cm) | 153.8 | 156.9 | 162.0 | 167.5 | 172.7 | 177.4 | 180.1 | 153.8 | 156.9 | 162.0 | 167.5 | 172.7 | 177.4 | 180.1 |
|  | 50th | 111.7 | 112.0 | 112.7 | 113.5 | 114.3 | 115.1 | 115.5 | 64.6 | 64.7 | 64.9 | 65.2 | 65.4 | 65.6 | 65.8 |
|  | 75th | 118.5 | 118.8 | 119.4 | 120.2 | 121.1 | 121.9 | 122.4 | 69.7 | 69.8 | 70.0 | 70.2 | 70.4 | 70.7 | 70.9 |
|  | 90th | 124.7 | 124.9 | 125.5 | 126.3 | 127.2 | 128.1 | 128.7 | 74.3 | 74.4 | 74.5 | 74.7 | 75.0 | 75.3 | 75.5 |
|  | 95th | 128.3 | 128.6 | 129.1 | 129.9 | 130.9 | 131.8 | 132.4 | 77.1 | 77.1 | 77.2 | 77.4 | 77.7 | 78.0 | 78.3 |
|  |  |  |  |  |  |  |  |  |  |  |  |  |  |  |  |
| 15 | height (in) | 62.6 | 63.8 | 65.7 | 67.8 | 69.8 | 71.5 | 72.5 | 62.6 | 63.8 | 65.7 | 67.8 | 69.8 | 71.5 | 72.5 |
|  | height (cm) | 159.0 | 162.0 | 166.9 | 172.2 | 177.2 | 181.6 | 184.2 | 159.0 | 162.0 | 166.9 | 172.2 | 177.2 | 181.6 | 184.2 |
|  | 50th | 113.8 | 114.2 | 114.8 | 115.7 | 116.5 | 117.2 | 117.7 | 65.3 | 65.4 | 65.6 | 65.9 | 66.1 | 66.4 | 66.5 |
|  | 75th | 120.8 | 121.1 | 121.7 | 122.6 | 123.4 | 124.3 | 124.8 | 70.6 | 70.6 | 70.8 | 71.1 | 71.3 | 71.6 | 71.8 |
|  | 90th | 127.1 | 127.3 | 127.9 | 128.8 | 129.7 | 130.6 | 131.1 | 75.3 | 75.3 | 75.5 | 75.7 | 76.0 | 76.3 | 76.5 |
|  | 95th | 130.9 | 131.1 | 131.7 | 132.5 | 133.4 | 134.4 | 135.0 | 78.1 | 78.1 | 78.3 | 78.5 | 78.8 | 79.1 | 79.3 |
|  |  |  |  |  |  |  |  |  |  |  |  |  |  |  |  |
| 16 | height (in) | 63.8 | 64.9 | 66.8 | 68.8 | 70.7 | 72.4 | 73.4 | 63.8 | 64.9 | 66.8 | 68.8 | 70.7 | 72.4 | 73.4 |
|  | height (cm) | 162.1 | 165.0 | 169.6 | 174.6 | 179.5 | 183.8 | 186.4 | 162.1 | 165.0 | 169.6 | 174.6 | 179.5 | 183.8 | 186.4 |
|  | 50th | 115.7 | 116.0 | 116.7 | 117.5 | 118.3 | 119.1 | 119.5 | 66.2 | 66.3 | 66.5 | 66.7 | 67.0 | 67.2 | 67.4 |
|  | 75th | 122.8 | 123.1 | 123.8 | 124.6 | 125.5 | 126.3 | 126.8 | 71.6 | 71.6 | 71.8 | 72.0 | 72.3 | 72.6 | 72.8 |
|  | 90th | 129.3 | 129.5 | 130.1 | 131.0 | 131.9 | 132.8 | 133.3 | 76.4 | 76.4 | 76.6 | 76.8 | 77.1 | 77.4 | 77.6 |
|  | 95th | 133.1 | 133.3 | 133.9 | 134.8 | 135.7 | 136.6 | 137.2 | 79.3 | 79.3 | 79.5 | 79.7 | 80.0 | 80.3 | 80.5 |
|  |  |  |  |  |  |  |  |  |  |  |  |  |  |  |  |
| 17 | height (in) | 64.5 | 65.5 | 67.3 | 69.2 | 71.1 | 72.8 | 73.8 | 64.5 | 65.5 | 67.3 | 69.2 | 71.1 | 72.8 | 73.8 |
|  | height (cm) | 163.8 | 166.5 | 170.9 | 175.8 | 180.7 | 184.9 | 187.5 | 163.8 | 166.5 | 170.9 | 175.8 | 180.7 | 184.9 | 187.5 |
|  | 50th | 117.0 | 117.3 | 118.0 | 118.8 | 119.7 | 120.4 | 120.9 | 67.2 | 67.3 | 67.5 | 67.7 | 68.0 | 68.2 | 68.4 |
|  | 75th | 124.3 | 124.6 | 125.2 | 126.1 | 127.0 | 127.8 | 128.3 | 72.7 | 72.8 | 73.0 | 73.2 | 73.5 | 73.7 | 73.9 |
|  | 90th | 130.9 | 131.2 | 131.8 | 132.6 | 133.5 | 134.4 | 135.0 | 77.7 | 77.7 | 77.9 | 78.1 | 78.4 | 78.7 | 78.9 |
|  | 95th | 134.9 | 135.1 | 135.7 | 136.5 | 137.5 | 138.4 | 139.0 | 80.7 | 80.7 | 80.8 | 81.1 | 81.4 | 81.7 | 81.9 |

**Supplementary Table S8. BP Levels for Girls by Age and Height Percentile**

| Age | **BP percentile** | **Systolic BP** | | | | | | | **Diastolic BP** | | | | | | |
| --- | --- | --- | --- | --- | --- | --- | --- | --- | --- | --- | --- | --- | --- | --- | --- |
|  |  | **Height Percentile** | | | | | | | **Height Percentile** | | | | | | |
|  |  | **5^th^** | **10^th^** | **25^th^** | **50^th^** | **75^th^** | **90^th^** | **95^th^** | **5th** | **10th** | **25th** | **50th** | **75th** | **90th** | **95th** |
| 3 | height (in) | 35.8 | 36.4 | 37.3 | 38.4 | 39.6 | 40.6 | 41.2 | 35.8 | 36.4 | 37.3 | 38.4 | 39.6 | 40.6 | 41.2 |
|  | height (cm) | 91.0 | 92.4 | 94.9 | 97.6 | 100.5 | 103.1 | 104.6 | 91.0 | 92.4 | 94.9 | 97.6 | 100.5 | 103.1 | 104.6 |
|  | 50th | 93.2 | 93.5 | 94.0 | 94.6 | 95.3 | 96.0 | 96.4 | 57.4 | 57.6 | 57.8 | 58.1 | 58.3 | 58.5 | 58.7 |
|  | 75th | 99.5 | 99.7 | 100.2 | 100.8 | 101.5 | 102.2 | 102.6 | 62.4 | 62.5 | 62.7 | 62.9 | 63.1 | 63.4 | 63.5 |
|  | 90th | 105.1 | 105.3 | 105.7 | 106.3 | 107.0 | 107.7 | 108.2 | 66.9 | 67.0 | 67.1 | 67.3 | 67.5 | 67.7 | 67.9 |
|  | 95th | 108.5 | 108.6 | 109.0 | 109.6 | 110.3 | 111.1 | 111.6 | 69.6 | 69.7 | 69.7 | 69.9 | 70.1 | 70.4 | 70.5 |
|  |  |  |  |  |  |  |  |  |  |  |  |  |  |  |  |
| 4 | height (in) | 38.3 | 38.9 | 39.9 | 41.1 | 42.4 | 43.5 | 44.2 | 38.3 | 38.9 | 39.9 | 41.1 | 42.4 | 43.5 | 44.2 |
|  | height (cm) | 97.2 | 98.7 | 101.4 | 104.5 | 107.6 | 110.5 | 112.2 | 97.2 | 98.7 | 101.4 | 104.5 | 107.6 | 110.5 | 112.2 |
|  | 50th | 94.4 | 94.7 | 95.2 | 95.8 | 96.5 | 97.2 | 97.6 | 58.0 | 58.2 | 58.4 | 58.7 | 58.9 | 59.1 | 59.3 |
|  | 75th | 100.6 | 100.8 | 101.3 | 101.9 | 102.6 | 103.3 | 103.8 | 63.0 | 63.1 | 63.2 | 63.4 | 63.7 | 63.9 | 64.1 |
|  | 90th | 106.2 | 106.4 | 106.8 | 107.4 | 108.1 | 108.8 | 109.3 | 67.4 | 67.4 | 67.5 | 67.7 | 68.0 | 68.2 | 68.4 |
|  | 95th | 109.6 | 109.7 | 110.1 | 110.7 | 111.4 | 112.2 | 112.7 | 70.0 | 70.1 | 70.1 | 70.3 | 70.5 | 70.8 | 71.0 |
|  |  |  |  |  |  |  |  |  |  |  |  |  |  |  |  |
| 5 | height (in) | 40.8 | 41.5 | 42.6 | 43.9 | 45.2 | 46.5 | 47.3 | 40.8 | 41.5 | 42.6 | 43.9 | 45.2 | 46.5 | 47.3 |
|  | height (cm) | 103.6 | 105.3 | 108.2 | 111.5 | 114.9 | 118.1 | 120.0 | 103.6 | 105.3 | 108.2 | 111.5 | 114.9 | 118.1 | 120.0 |
|  | 50th | 95.7 | 96.0 | 96.5 | 97.1 | 97.8 | 98.5 | 98.9 | 58.7 | 58.8 | 59.0 | 59.3 | 59.5 | 59.8 | 59.9 |
|  | 75th | 101.9 | 102.1 | 102.6 | 103.2 | 103.9 | 104.6 | 105.1 | 63.5 | 63.6 | 63.8 | 64.0 | 64.2 | 64.5 | 64.6 |
|  | 90th | 107.5 | 107.6 | 108.1 | 108.6 | 109.3 | 110.1 | 110.6 | 67.9 | 67.9 | 68.1 | 68.2 | 68.5 | 68.7 | 68.9 |
|  | 95th | 110.8 | 111.0 | 111.3 | 111.9 | 112.6 | 113.4 | 113.9 | 70.5 | 70.5 | 70.6 | 70.8 | 71.0 | 71.3 | 71.5 |
|  |  |  |  |  |  |  |  |  |  |  |  |  |  |  |  |
| 6 | height (in) | 43.3 | 44.0 | 45.2 | 46.6 | 48.1 | 49.4 | 50.3 | 43.3 | 44.0 | 45.2 | 46.6 | 48.1 | 49.4 | 50.3 |
|  | height (cm) | 110.0 | 111.8 | 114.9 | 118.4 | 122.1 | 125.6 | 127.7 | 110.0 | 111.8 | 114.9 | 118.4 | 122.1 | 125.6 | 127.7 |
|  | 50th | 97.1 | 97.4 | 97.9 | 98.5 | 99.2 | 99.9 | 100.3 | 59.4 | 59.5 | 59.7 | 60.0 | 60.2 | 60.4 | 60.6 |
|  | 75th | 103.3 | 103.5 | 104.0 | 104.6 | 105.3 | 106.0 | 106.5 | 64.2 | 64.2 | 64.4 | 64.6 | 64.9 | 65.1 | 65.3 |
|  | 90th | 108.8 | 109.0 | 109.5 | 110.0 | 110.7 | 111.5 | 112.0 | 68.5 | 68.5 | 68.6 | 68.8 | 69.1 | 69.3 | 69.5 |
|  | 95th | 112.2 | 112.4 | 112.7 | 113.3 | 114.0 | 114.8 | 115.3 | 71.1 | 71.1 | 71.2 | 71.3 | 71.6 | 71.8 | 72.0 |
|  |  |  |  |  |  |  |  |  |  |  |  |  |  |  |  |
| 7 | height (in) | 45.6 | 46.4 | 47.7 | 49.2 | 50.7 | 52.2 | 53.0 | 45.6 | 46.4 | 47.7 | 49.2 | 50.7 | 52.2 | 53.0 |
|  | height (cm) | 115.9 | 117.8 | 121.1 | 124.9 | 128.8 | 132.5 | 134.7 | 115.9 | 117.8 | 121.1 | 124.9 | 128.8 | 132.5 | 134.7 |
|  | 50th | 98.6 | 98.9 | 99.4 | 100.0 | 100.7 | 101.4 | 101.8 | 60.0 | 60.2 | 60.4 | 60.7 | 60.9 | 61.1 | 61.3 |
|  | 75th | 104.8 | 105.0 | 105.5 | 106.1 | 106.8 | 107.5 | 108.0 | 64.8 | 64.9 | 65.1 | 65.3 | 65.5 | 65.8 | 65.9 |
|  | 90th | 110.4 | 110.6 | 111.0 | 111.6 | 112.3 | 113.0 | 113.5 | 69.1 | 69.2 | 69.3 | 69.5 | 69.7 | 70.0 | 70.2 |
|  | 95th | 113.7 | 113.9 | 114.3 | 114.8 | 115.5 | 116.3 | 116.8 | 71.7 | 71.7 | 71.8 | 72.0 | 72.2 | 72.5 | 72.7 |
| 8 | height (in) | 47.6 | 48.4 | 49.8 | 51.4 | 53.0 | 54.5 | 55.5 | 47.6 | 48.4 | 49.8 | 51.4 | 53.0 | 54.5 | 55.5 |
|  | height (cm) | 121.0 | 123.0 | 126.5 | 130.6 | 134.7 | 138.5 | 140.9 | 121.0 | 123.0 | 126.5 | 130.6 | 134.7 | 138.5 | 140.9 |
|  | 50th | 100.2 | 100.4 | 101.0 | 101.6 | 102.3 | 103.0 | 103.4 | 60.8 | 60.9 | 61.1 | 61.4 | 61.6 | 61.8 | 62.0 |
|  | 75th | 106.4 | 106.6 | 107.1 | 107.7 | 108.4 | 109.1 | 109.6 | 65.5 | 65.6 | 65.8 | 66.0 | 66.2 | 66.5 | 66.7 |
|  | 90th | 112.0 | 112.2 | 112.6 | 113.2 | 113.9 | 114.6 | 115.1 | 69.8 | 69.8 | 70.0 | 70.2 | 70.4 | 70.7 | 70.9 |
|  | 95th | 115.4 | 115.5 | 115.9 | 116.5 | 117.2 | 117.9 | 118.5 | 72.4 | 72.4 | 72.5 | 72.7 | 72.9 | 73.2 | 73.4 |
|  |  |  |  |  |  |  |  |  |  |  |  |  |  |  |  |
| 9 | height (in) | 49.3 | 50.2 | 51.7 | 53.4 | 55.1 | 56.7 | 57.7 | 49.3 | 50.2 | 51.7 | 53.4 | 55.1 | 56.7 | 57.7 |
|  | height (cm) | 125.3 | 127.6 | 131.3 | 135.6 | 140.1 | 144.1 | 146.6 | 125.3 | 127.6 | 131.3 | 135.6 | 140.1 | 144.1 | 146.6 |
|  | 50th | 101.8 | 102.1 | 102.6 | 103.2 | 103.9 | 104.6 | 105.0 | 61.5 | 61.6 | 61.8 | 62.1 | 62.3 | 62.6 | 62.7 |
|  | 75th | 108.1 | 108.3 | 108.8 | 109.4 | 110.1 | 110.8 | 111.2 | 66.2 | 66.3 | 66.5 | 66.7 | 67.0 | 67.2 | 67.4 |
|  | 90th | 113.7 | 113.9 | 114.3 | 114.9 | 115.6 | 116.4 | 116.8 | 70.5 | 70.6 | 70.7 | 70.9 | 71.2 | 71.4 | 71.6 |
|  | 95th | 117.1 | 117.3 | 117.7 | 118.2 | 118.9 | 119.7 | 120.2 | 73.1 | 73.1 | 73.2 | 73.4 | 73.7 | 73.9 | 74.1 |
|  |  |  |  |  |  |  |  |  |  |  |  |  |  |  |  |
| 10 | height (in) | 51.1 | 52.0 | 53.7 | 55.5 | 57.4 | 59.1 | 60.2 | 51.1 | 52.0 | 53.7 | 55.5 | 57.4 | 59.1 | 60.2 |
|  | height (cm) | 129.7 | 132.2 | 136.3 | 141.0 | 145.8 | 150.2 | 152.8 | 129.7 | 132.2 | 136.3 | 141.0 | 145.8 | 150.2 | 152.8 |
|  | 50th | 103.4 | 103.7 | 104.2 | 104.9 | 105.6 | 106.2 | 106.7 | 62.2 | 62.3 | 62.6 | 62.8 | 63.1 | 63.3 | 63.4 |
|  | 75th | 109.8 | 110.0 | 110.5 | 111.1 | 111.8 | 112.5 | 113.0 | 67.0 | 67.1 | 67.3 | 67.5 | 67.7 | 68.0 | 68.2 |
|  | 90th | 115.5 | 115.7 | 116.1 | 116.7 | 117.4 | 118.1 | 118.6 | 71.3 | 71.4 | 71.5 | 71.7 | 72.0 | 72.2 | 72.4 |
|  | 95th | 118.9 | 119.1 | 119.5 | 120.0 | 120.7 | 121.5 | 122.0 | 73.9 | 73.9 | 74.0 | 74.2 | 74.5 | 74.8 | 75.0 |
|  |  |  |  |  |  |  |  |  |  |  |  |  |  |  |  |
| 11 | height (in) | 53.4 | 54.5 | 56.2 | 58.2 | 60.2 | 61.9 | 63.0 | 53.4 | 54.5 | 56.2 | 58.2 | 60.2 | 61.9 | 63.0 |
|  | height (cm) | 135.6 | 138.3 | 142.8 | 147.8 | 152.8 | 157.3 | 160.0 | 135.6 | 138.3 | 142.8 | 147.8 | 152.8 | 157.3 | 160.0 |
|  | 50th | 105.1 | 105.3 | 105.9 | 106.5 | 107.2 | 107.9 | 108.3 | 62.9 | 63.0 | 63.3 | 63.5 | 63.8 | 64.0 | 64.1 |
|  | 75th | 111.5 | 111.7 | 112.2 | 112.8 | 113.5 | 114.2 | 114.7 | 67.8 | 67.8 | 68.0 | 68.3 | 68.5 | 68.8 | 68.9 |
|  | 90th | 117.3 | 117.5 | 117.9 | 118.4 | 119.2 | 119.9 | 120.4 | 72.1 | 72.2 | 72.3 | 72.5 | 72.8 | 73.1 | 73.2 |
|  | 95th | 120.7 | 120.9 | 121.3 | 121.8 | 122.5 | 123.3 | 123.8 | 74.7 | 74.8 | 74.9 | 75.1 | 75.3 | 75.6 | 75.8 |
|  |  |  |  |  |  |  |  |  |  |  |  |  |  |  |  |
| 12 | height (in) | 56.2 | 57.3 | 59.0 | 60.9 | 62.8 | 64.5 | 65.5 | 56.2 | 57.3 | 59.0 | 60.9 | 62.8 | 64.5 | 65.5 |
|  | height (cm) | 142.8 | 145.5 | 149.9 | 154.8 | 159.6 | 163.8 | 166.4 | 142.8 | 145.5 | 149.9 | 154.8 | 159.6 | 163.8 | 166.4 |
|  | 50th | 106.6 | 106.9 | 107.4 | 108.0 | 108.7 | 109.4 | 109.8 | 63.6 | 63.8 | 64.0 | 64.2 | 64.5 | 64.7 | 64.8 |
|  | 75th | 113.1 | 113.4 | 113.8 | 114.4 | 115.1 | 115.8 | 116.3 | 68.5 | 68.6 | 68.8 | 69.0 | 69.3 | 69.5 | 69.7 |
|  | 90th | 119.0 | 119.2 | 119.6 | 120.2 | 120.9 | 121.6 | 122.1 | 72.9 | 73.0 | 73.2 | 73.4 | 73.6 | 73.9 | 74.1 |
|  | 95th | 122.5 | 122.7 | 123.1 | 123.6 | 124.3 | 125.1 | 125.6 | 75.6 | 75.6 | 75.8 | 75.9 | 76.2 | 76.5 | 76.7 |
|  |  |  |  |  |  |  |  |  |  |  |  |  |  |  |  |
| 13 | height (in) | 58.3 | 59.3 | 60.9 | 62.7 | 64.5 | 66.1 | 67.0 | 58.3 | 59.3 | 60.9 | 62.7 | 64.5 | 66.1 | 67.0 |
|  | height (cm) | 148.1 | 150.6 | 154.7 | 159.2 | 163.7 | 167.8 | 170.2 | 148.1 | 150.6 | 154.7 | 159.2 | 163.7 | 167.8 | 170.2 |
|  | 50th | 108.0 | 108.3 | 108.8 | 109.5 | 110.2 | 110.8 | 111.3 | 64.3 | 64.4 | 64.7 | 64.9 | 65.2 | 65.4 | 65.5 |
|  | 75th | 114.7 | 114.9 | 115.4 | 116.0 | 116.7 | 117.4 | 117.8 | 69.3 | 69.4 | 69.6 | 69.8 | 70.1 | 70.3 | 70.5 |
|  | 90th | 120.6 | 120.8 | 121.2 | 121.8 | 122.5 | 123.3 | 123.8 | 73.8 | 73.9 | 74.0 | 74.2 | 74.5 | 74.8 | 75.0 |
|  | 95th | 124.2 | 124.4 | 124.8 | 125.3 | 126.0 | 126.8 | 127.3 | 76.5 | 76.5 | 76.7 | 76.9 | 77.1 | 77.4 | 77.6 |
|  |  |  |  |  |  |  |  |  |  |  |  |  |  |  |  |
| 14 | height (in) | 59.3 | 60.2 | 61.8 | 63.5 | 65.2 | 66.8 | 67.7 | 59.3 | 60.2 | 61.8 | 63.5 | 65.2 | 66.8 | 67.7 |
|  | height (cm) | 150.6 | 153.0 | 156.9 | 161.3 | 165.7 | 169.7 | 172.1 | 150.6 | 153.0 | 156.9 | 161.3 | 165.7 | 169.7 | 172.1 |
|  | 50th | 109.3 | 109.5 | 110.1 | 110.7 | 111.4 | 112.1 | 112.5 | 65.0 | 65.1 | 65.3 | 65.6 | 65.8 | 66.1 | 66.2 |
|  | 75th | 116.0 | 116.3 | 116.7 | 117.3 | 118.0 | 118.7 | 119.2 | 70.1 | 70.2 | 70.4 | 70.6 | 70.9 | 71.1 | 71.3 |
|  | 90th | 122.1 | 122.3 | 122.7 | 123.3 | 124.0 | 124.7 | 125.2 | 74.6 | 74.7 | 74.9 | 75.1 | 75.4 | 75.7 | 75.8 |
|  | 95th | 125.7 | 125.9 | 126.3 | 126.9 | 127.6 | 128.3 | 128.8 | 77.4 | 77.4 | 77.6 | 77.8 | 78.1 | 78.4 | 78.6 |
|  |  |  |  |  |  |  |  |  |  |  |  |  |  |  |  |
| 15 | height (in) | 59.7 | 60.6 | 62.2 | 63.9 | 65.6 | 67.2 | 68.1 | 59.7 | 60.6 | 62.2 | 63.9 | 65.6 | 67.2 | 68.1 |
|  | height (cm) | 151.7 | 154.0 | 157.9 | 162.3 | 166.7 | 170.6 | 173.0 | 151.7 | 154.0 | 157.9 | 162.3 | 166.7 | 170.6 | 173.0 |
|  | 50th | 110.2 | 110.5 | 111.0 | 111.7 | 112.4 | 113.0 | 113.5 | 65.6 | 65.7 | 66.0 | 66.2 | 66.5 | 66.7 | 66.8 |
|  | 75th | 117.1 | 117.4 | 117.8 | 118.4 | 119.1 | 119.9 | 120.3 | 70.8 | 70.9 | 71.1 | 71.4 | 71.6 | 71.9 | 72.0 |
|  | 90th | 123.3 | 123.6 | 124.0 | 124.5 | 125.3 | 126.0 | 126.5 | 75.5 | 75.6 | 75.7 | 76.0 | 76.2 | 76.5 | 76.7 |
|  | 95th | 127.1 | 127.2 | 127.6 | 128.2 | 128.9 | 129.7 | 130.2 | 78.3 | 78.4 | 78.5 | 78.7 | 79.0 | 79.3 | 79.5 |
|  |  |  |  |  |  |  |  |  |  |  |  |  |  |  |  |
| 16 | height (in) | 59.9 | 60.8 | 62.4 | 64.1 | 65.8 | 67.4 | 68.3 | 59.9 | 60.8 | 62.4 | 64.1 | 65.8 | 67.4 | 68.3 |
|  | height (cm) | 152.1 | 154.5 | 158.4 | 162.8 | 167.1 | 171.1 | 173.4 | 152.1 | 154.5 | 158.4 | 162.8 | 167.1 | 171.1 | 173.4 |
|  | 50th | 110.9 | 111.2 | 111.7 | 112.3 | 113.0 | 113.7 | 114.1 | 66.2 | 66.3 | 66.6 | 66.8 | 67.1 | 67.3 | 67.4 |
|  | 75th | 117.9 | 118.2 | 118.6 | 119.2 | 119.9 | 120.7 | 121.1 | 71.6 | 71.7 | 71.9 | 72.1 | 72.4 | 72.6 | 72.8 |
|  | 90th | 124.3 | 124.5 | 124.9 | 125.5 | 126.2 | 126.9 | 127.4 | 76.4 | 76.4 | 76.6 | 76.8 | 77.1 | 77.4 | 77.6 |
|  | 95th | 128.1 | 128.3 | 128.7 | 129.2 | 129.9 | 130.7 | 131.2 | 79.2 | 79.3 | 79.4 | 79.7 | 80.0 | 80.3 | 80.5 |
|  |  |  |  |  |  |  |  |  |  |  |  |  |  |  |  |
| 17 | height (in) | 60.0 | 60.9 | 62.5 | 64.2 | 65.9 | 67.5 | 68.4 | 60.0 | 60.9 | 62.5 | 64.2 | 65.9 | 67.5 | 68.4 |
|  | height (cm) | 152.4 | 154.7 | 158.7 | 163.0 | 167.4 | 171.3 | 173.7 | 152.4 | 154.7 | 158.7 | 163.0 | 167.4 | 171.3 | 173.7 |
|  | 50th | 111.1 | 111.4 | 111.9 | 112.5 | 113.2 | 113.9 | 114.3 | 66.8 | 66.9 | 67.1 | 67.4 | 67.6 | 67.9 | 68.0 |
|  | 75th | 118.3 | 118.6 | 119.0 | 119.6 | 120.3 | 121.0 | 121.5 | 72.3 | 72.4 | 72.6 | 72.8 | 73.1 | 73.3 | 73.5 |
|  | 90th | 124.8 | 125.0 | 125.4 | 126.0 | 126.7 | 127.5 | 127.9 | 77.2 | 77.3 | 77.4 | 77.7 | 78.0 | 78.2 | 78.4 |
|  | 95th | 128.7 | 128.9 | 129.3 | 129.8 | 130.6 | 131.3 | 131.8 | 80.1 | 80.2 | 80.4 | 80.6 | 80.9 | 81.2 | 81.4 |

**References**

1. The fourth report on the diagnosis, evaluation, and treatment of high blood pressure in children and adolescents. Pediatrics. 2004;114(2 Suppl 4th Report):555-76.

2. Flynn JT, Kaelber DC, Baker-Smith CM, Blowey D, Carroll AE, Daniels SR, et al. Clinical Practice Guideline for Screening and Management of High Blood Pressure in Children and Adolescents. Pediatrics. 2017;140(3).

3. Muntner P, Shimbo D, Carey RM, Charleston JB, Gaillard T, Misra S, et al. Measurement of Blood Pressure in Humans: A Scientific Statement From the American Heart Association. Hypertension. 2019;73(5):e35-e66.

4. Rabi DM, McBrien KA, Sapir-Pichhadze R, Nakhla M, Ahmed SB, Dumanski SM, et al. Hypertension Canada's 2020 Comprehensive Guidelines for the Prevention, Diagnosis, Risk Assessment, and Treatment of Hypertension in Adults and Children. Can J Cardiol. 2020;36(5):596-624.

5. Unger T, Borghi C, Charchar F, Khan NA, Poulter NR, Prabhakaran D, et al. 2020 International Society of Hypertension Global Hypertension Practice Guidelines. Hypertension. 2020;75(6):1334-57.

6. de Simone G, Mancusi C, Hanssen H, Genovesi S, Lurbe E, Parati G, et al. Hypertension in children and adolescents. European heart journal. 2022.

7. Rosner B, Cook N, Portman R, Daniels S, Falkner B. Determination of blood pressure percentiles in normal-weight children: some methodological issues. American journal of epidemiology. 2008;167(6):653-66.

8. Bates D MM, Bolker B, Walker S Fitting Linear Mixed-Effects Models Using lme4. Journal of Statistical Software. 2015;67(1):1-48.
